# A repository for the publication and sharing of heterogeneous materials data

**DOI:** 10.1038/s41597-022-01897-z

**Published:** 2022-12-27

**Authors:** Haiyan Gong, Jie He, Xiaotong Zhang, Lei Duan, Ziqi Tian, Wei Zhao, Fuzhou Gong, Tong Liu, Zongguo Wang, Haifeng Zhao, Weipeng Jia, Lei Zhang, Xue Jiang, Wencong Chen, Shilong Liu, Hao Xiu, Wenjin Yang, Jiawang Wan

**Affiliations:** 1 Beijing Advanced Innovation Center for Materials Genome Engineering, School of Computer and Communication Engineering, University of Science and Technology Beijing, Beijing, 100083 China; 2 Beijing Key Laboratory of Knowledge Engineering for Materials Science, University of Science and Technology Beijing, Beijing, 100083 China; 3 Shunde Innovation School, University of Science and Technology Beijing, Foshan, 528399 China; 4 Wangjiang Campus, Sichuan University, Wuhou, Chengdu, Sichuan Province P. R. China; 5 Ningbo Institute of Materials Technology and Engineering, Chinese Academy of Sciences, Ningbo, 315201 Zhejiang China; 6 General Data Technology Co., Ltd., East Tower of Putian Innovation Industrial Park 22 Kaihua Road, Huayuan Industrial Park high-tech zone, Tianjin, China; 7 CEMS, NCMIS, RCSDS, Academy of Mathematics and Systems Science, Chinese Academy of Sciences, Beijing, 100190 China; 8 University of Chinese Academy of Sciences, Beijing, 100049 China; 9 Beijing Computing Center Co., Ltd, No.3 Beike Industry Base, No.7 Mid, Fengxian Rd, Haidian District, Beijing, China; 10 Computer Network Information Center, Chinese Academy of Sciences, Beijing, 100190 China; 11 Beijing Synchrotron Radiation Facility, Institute of High Energy Physics, Chinese Academy of Sciences, 19B Yuquan Road, Shijingshan District, 100049 Beijing, P. R. China

**Keywords:** Research management, Computational methods

## Abstract

National Materials Data Management and Service platform (NMDMS) is a materials data repository for the publication and sharing of heterogeneous materials scientific data and follows the FAIR principles: Findable, Accessible, Interoperable, and Reusable. To ensure data are ‘Interoperable, NMDMS uses a user-friendly semi-structured scientific data model, named dynamic container’, to define, exchange, and store heterogeneous scientific data. Then, a personalized yet standardized data submission subsystem, a rigorous project data review and publication subsystem, and a multi-granularity data query and retrieval subsystem collaboratively make data ‘Reusable’, ‘Findable’, and ‘Accessible’. Finally, China’s “National Key R&D Program: Material Genetic Engineering Key Special Project” has adopted NMDMS to publish and share its project data. There are 12,251,040 pieces of data published in NMDMS since 2018, under 87 categories and 1,912 user-defined schemas from 45 projects. The platform has been accessed 908875 times, and 2403,208 pieces of data have been downloaded. In short, NMDMS effectively accelerates the publication and sharing of material project data in China.

## Introduction

With the boom of materials design based on artificial intelligence and material science data, material scientific data is becoming a factor of production, a national strategic resource, and a focus of international materials technology competition. It is therefore crucial for both countries and material R & D institutions to develop a platform for collecting, storing, publishing, and sharing valuable material scientific data produced by the research activities, such as experiments, simulation, calculation, and manufacturing.

The material scientific data is featured with many categories, attributes, and strong professionalism. In every scientific research activity, data is generated and used in a highly personalized manner. To meet the requirement of personalization, the data platform has to be able to customize data schemas, and store, display, find and share the heterogeneous data. On the one hand, it should be a personalized heterogeneous data publication and sharing platform that supports the FAIR (‘Findable’, ‘Accessible’, ‘Interoperable’, and ‘Reusable’) principle described in Supplementary Table S1. On the other hand, the user interface of the data platform has to be friendly to the material researchers and special consideration should be given to their lack of professional database management skills. Combining these requirements leads to huge challenges in developing a generic material scientific data platform.

All scientific data consists of metadata and data content, where the metadata generally exists in a data form with a fixed structure. However, the data content mainly exists in available material data platforms in three forms: structured, non-structured, and semi-structured. The representative platform using the first form (*i.e*., structured form, Supplementary Table S2) aims to create a specific material domain data platform with defined materials properties fields that users can use, such as the Inorganic Crystal Structure Database (ICSD)^1^, the computational database (*e.g*., the Materials Project^2^, matcloud^3^, AFLOWLIB^4^, NOMAD^5^, Harvard Clean Energy Project^6^), chemical database (*e.g*., ChemSpider^7^, MPDS^8^, PubChem^9^), Crystallography database (*e.g*., Cambridge Crystallographic Data Centre (CCDC)^10^, CrystMet^11^, Crystallography Open Database (CoD)^12^, Powder Diffraction File (PDF)^13^) the National Material Environmental Corrosion Platform^14^, 3D Materials Atlas^15^, American Mineralogist Crystal Structure Database^16^, ASM Alloy Center Database^17^, ASM Phase Diagrams^17^, and so on. This form cannot meet the requirement of data structure customization, and it makes it low efficient to collect multi-domain data.

The second form (*i.e*., non-structured form, Supplementary Table S3) stores the data content as attachments or data accession URLs of other data repositories. Such as Materials Commons^18^, NMRR^19^, DSpace^20^, Dryad^21^, Figshare^22^, Mendeley Data^23^, Zenodo^24^, DataHub^25^, DANS, EUDat^26^, Material Data Facility^27^, AIST^28^, MatWeb^29^, Granta CES Selector^30^, Knovel^31^, MATDAT^32^, NIST Standard Reference Data^33^, Pauling File^34^, SpringerMaterials^35^, Total Materia^36^, and other scientific datasets retrieval platforms that are not domain-specific, including Data Citation Index^37^, DataCite Search^38,39^, DataMed^40^, and Dataset Search^41^. In exchange for being more versatile and friendly to the data providers, this form is not capable of querying data content. Therefore, it is challenging for the second type to meet the ‘Findable’, ‘Interoperable’, and ‘Reusable’ of the FAIR principle^42^ for scientific data.

The semi-structured form begins to be popularly used in storing the data content to overcome the drawbacks of the aforementioned two forms. The representative works include Materials Data Curation System^43^ (MDCS), Materials Cloud^44^, and our previous work, Materials Genome Engineering Databases^45^ (MGED). These platforms mainly store the data in documents (*e.g*., XML and JSON) and provide a visual data schema construction interface for users to define their personalized data structure. By using the third form, users can submit their personalized data without having to consult with database managers to design the data fields.

On the other hand, since most scientific data are generated from scientific research under a project, many foundations and countries require projects to share data to reuse data, decreasing the waste of data sources and accelerating the research process. For example, the national science foundation^46^ (NSF) requires principal investigators (PIs) to provide data management plans. European Union and China published scientific data management and open-sharing policies to submit project-related data in a universal data platform^47,48^. The publication and sharing of scientific data have gradually become factors affecting project performance evaluation^49^. To support the project data publication and sharing, some material platforms, such as Citrination^43^ and Materials Commons^18^, require users to create a team or project to submit data under a team or project to meet the requirement of scientific data management and open sharing policies. However, these platforms do not provide a data review process to publish data and may result in the generation of low-quality scientific data. Therefore, a platform that can simultaneously publish, share, and evaluate project scientific data is urgently needed to accelerate sharing of high-quality scientific research data.

Considering the above two requirements (a generical material scientific data platform that can publish, share, and evaluate project scientific data), our previous work MGED was selected as the storage solution to build the national material data management system (NMDMS) in China for the project scientific data publication and sharing of National key R&D plan material genetic engineering key special projects. The exchange and storage model for heterogeneous scientific data was defined based on MGED to ensure data is ‘Interoperable’. Based on the model, we designed a personalized and standardized data submission subsystem, a rigorous project scientific data review and publication subsystem, and a multi-granularity data query and acquisition subsystem to achieve data ‘Reusable’, ‘Findable’, and ‘Accessible’. Up to 2022, NMDMS has published 12,251,040 data records from 87 material categories and 45 projects. There have been 908875 visits to NMDMS, and 2403,208 pieces of data have been downloaded. As demonstrated by these records, NMDMS is an effective generic materials platform that aims for publishing and sharing personalized and standardized materials data.

## Results

**Fig. 1:**
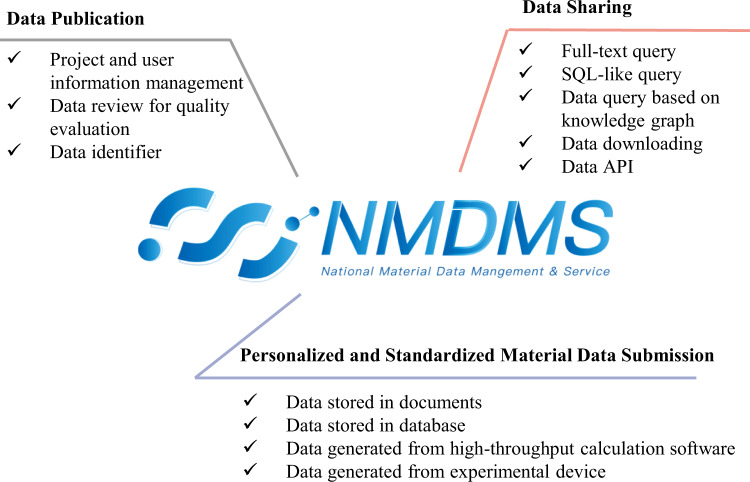
NMDMS is mainly divided into three systems: Personalized and Standardized Material Data Submission, Data Publication, and Data Sharing.

NMDMS aims to provide a general material data repository to publish and share heterogeneous material scientific data and follows the FAIR principles described in Supplementary Table S1. As part of the NMDMS, three systems are included: the Data Submission System, the Data Publication System, and the Data Sharing System. Fig. 1 illustrates how the three systems of NMDMS mirror the scientific data cycle, beginning with “data submission” and extending to “data publication”, and “data sharing” for data application.

Specifically, NMDMS is a general repository where researchers can submit data in various formats, including personalized and standardized data via the Data Submission System. Data submitted in NMDMS would be reviewed through a rigorous process via the Data Publication System to guarantee data quality. Finally, reviewed data could be published and queried by three types of query methods via the Data Sharing System. Below, we provide a detailed description of the three systems.

### Personalized and standardized material data submission

**Fig. 2:**
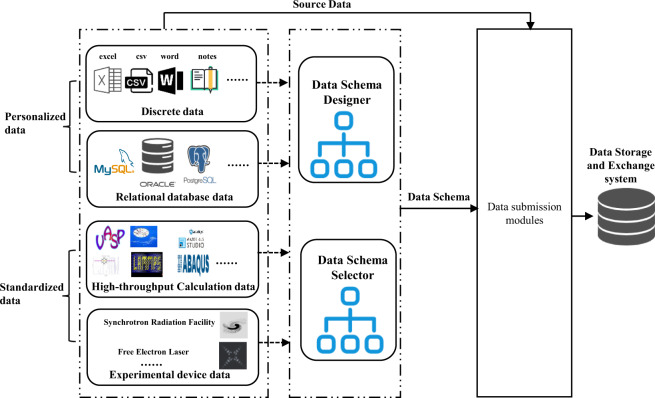
The description and data submission methods of the four types of material data.

This section provides an overview of the material data submission system (DSS). The scientific data exists in two forms (Fig. 2): one form is personalized data stored in a document (EXCEL, CSV, WORD, notes) or databases, which is usually generated by users during their daily experiments. Another form is standardized bulk data generated from calculation software or experimental device data, which can be further interpreted. DSS is designed to collect data based on the data storage and exchange system (DSES).

Before submitting data, a data schema is required. For personalized data, NMDMS provides a graphical data schema designer. For standardized data, the system administrator designs standardized schemas in advance, and users select standardized data schemas. Then, four submission modules, including a discrete data submission module, relational database data submission module, high-throughput calculation data submission module, and experiment device data submission module are provided for users to submit personalized and standardized data. Each module can automatically or manually extract, transform, and load source data into DSES based on the data schema. In the following, we focus on the description of the data schema designer and submission modules.

### Data schema designer

Data schema-designer provides a comprehensible, easy-to-operate, graphical data schema editor for material researchers to design multi-domain data schemas without considering how to use databases to create personalized data schema and design submission pages for multi-domain data. This can significantly reduce the time to build a general, multi-domain database with a specialized data schema.

**Fig. 3:**
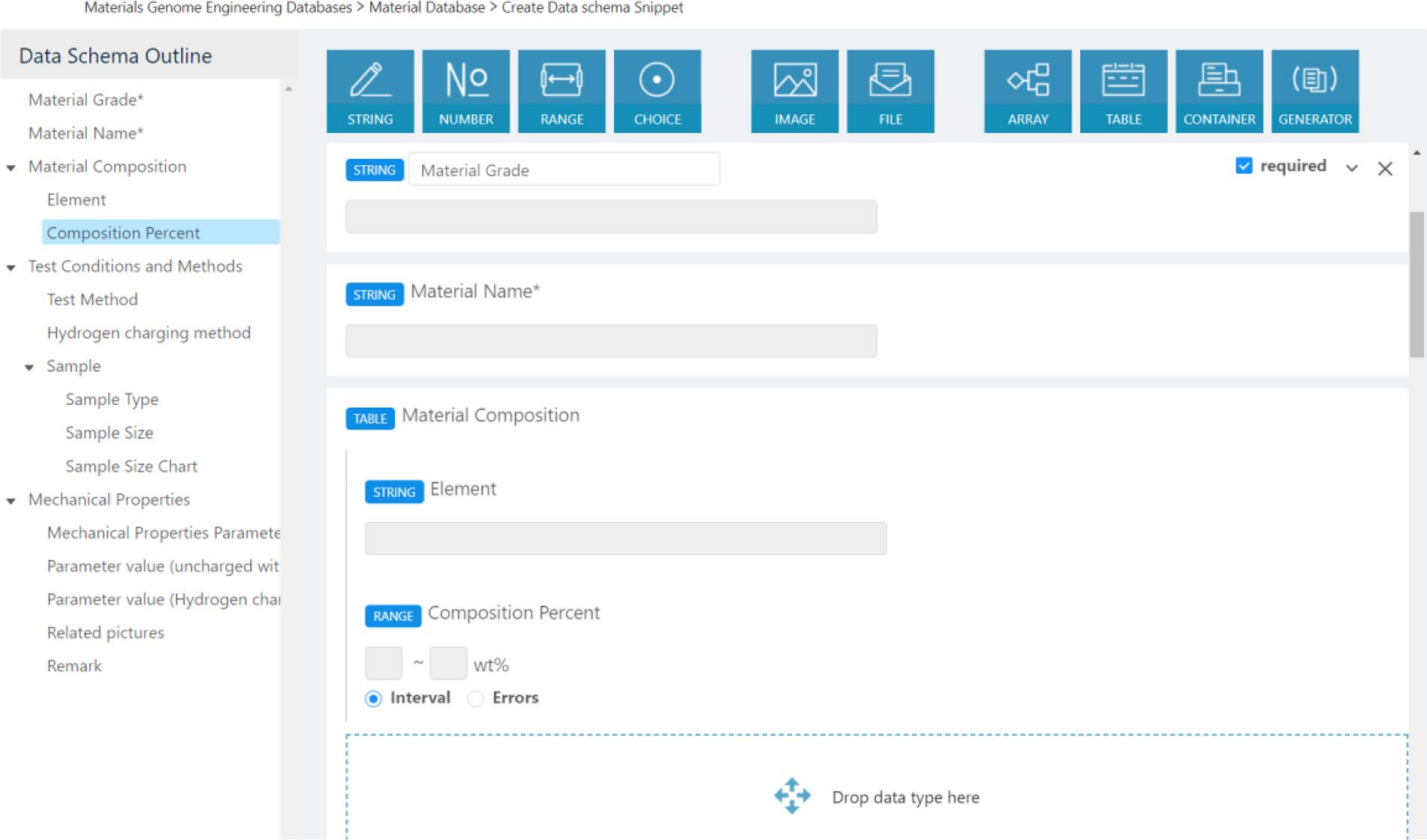
An example of designing hydrogen embrittlement experimental data schema by just dragging icons of the ten data types.

As Fig. 3 shows, the data schema designer provides a what-you-see-is-what-you-get graphical user interface to design material data’s personalized attributes and structures by just dragging and dropping icons of the ten easy-to-understand data types. Among the ten data types, primitive data types including string, number, range, choice, image, and file are designed to define data attributes, and composite data types including array, table, container, and generator are designed to organize the data structure. The data schema structure is displayed clearly in the form of a directory to help users understand the data organization.

The data schema designer is flexible and easy to operate. By dragging and dropping the ten data icons or data schema outline on the left, data schemas can be easily created or modified. ‘Importing existing data schema’ allows users to load existing standardized data schema to learn how to organize data schema and edit it to create their customized schema. The user-defined schema makes it easier for non-experts to use but may lead to difficulty in interoperating with other similar data. Overemphasis on data operability may lead to a lack of standard data. However, If the data format is standardized and fixed, users will be limited to the platform format, reducing the ease of use of the platform. Therefore, the data schema designer module makes a compromise between user ease of use and data standard. Firstly, a ‘Data schema snippet’ is provided to design general data schema, such as material composition, that can be reloaded into any other data schema by simply clicking on the dotted box. The ‘data schema snippet’ can reduce the time to build a general, standardized data schema repeatedly. Secondly, with the built-in materials lexicon included in the data schema designer, users can easily create the standardized data schema by searching for standardized data fields. The book of materials comprehensive dictionary and approved data schemas are used to generate the materials lexicon. Details on creating a dynamic container schema can be seen on YouTube https://www.youtube.com/watch?v=G0_54ST97Ns.

### Data submission module

Four modules are provided to facilitate transforming source data into DSES according to the four types of data characteristics. For data with a fixed format, such as high-throughput calculation data and experiment device data, NMDMS provides standardized data schemas to minimize the submission of irregular data. For personalized data, graphical ETL tools can reduce the time to convert source data to DSES format data.

The discrete data submission module requires the data providers to enter data one by one through the web page or manually organize the data into EXCEL (or JSON, or XML format) with data schema and upload them in batches. Data with attachments can also be parsed from compressed files in excel format file. It is suitable for submitting experimental data stored in files, such as word, excel, and so on. Using the discrete data submission module, we are able to store any information about the data as long as users define the corresponding fields in data schemas (e.g., instrument settings, synthesis methods, simulation input parameters). We can also use third-party interfaces and meticulously pre-designed schemas to capture data generated by devices used in experiments.

The relational database data submission module provides two ETL tools, including GBase Migration Toolkit and GBase2DCD Migration Toolkit, to extract, transform, and load relational database data into DSES. Firstly, GBase Migration Toolkit transforms heterogeneous source relational database data (the supported databases are listed in Table 1) into a relational database GBase. Then, GBase2DCD Migration Toolkit converts GBase database data into DSES by mapping the data fields created by the data schema designer. The graphical data migration tool can reduce the time to convert database data without programming.

**Table 1:** The supported data types of four data submission modules.

data submission module	Supported data type
Discrete data submission module	Excel, XML, JSON
Relational database data submission module	ACCESS, Oracle, SQL Server2005, DM, DB2, MySQL, ShenTong, GBase8sV8.3, GBase8t, GBase8s, PostgreSQL, and Teradata
High-throughput calculation data submission module	VASP, ABINIT and LAMMPS
Experiment device data submission module	synchrotron radiation devices, spallation neutron sources, and free electron lasers

**Fig. 4:**
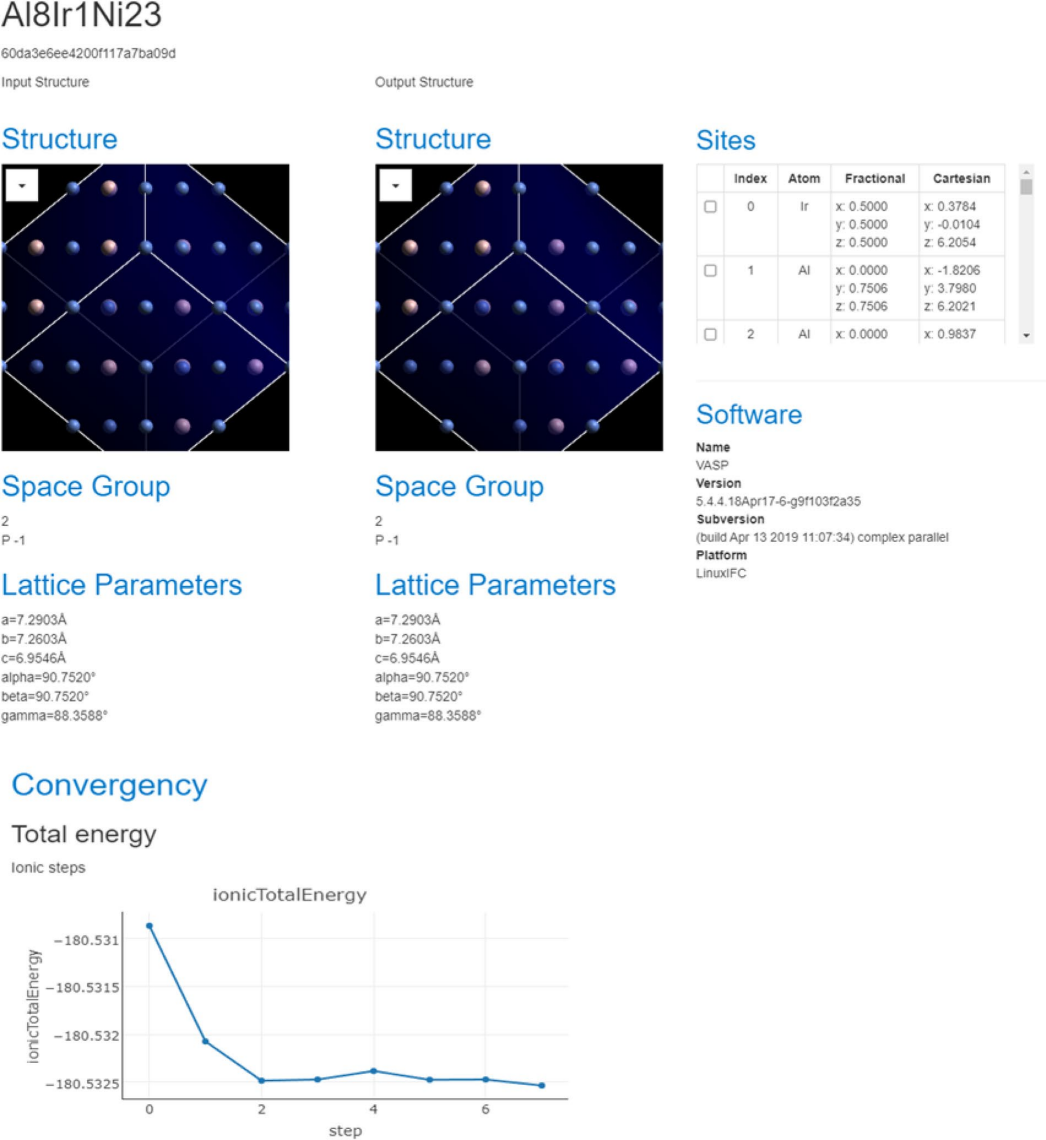
Data extraction and visualization for data from VASP software. Lattice structure, space group, lattice parameters, sites, software, and the convergency curve of the total ionic energy are all displayed.

The High-throughput calculation data submission module and experiment device data submission module aim to collect standardized data from calculation software or experiment device, supporting single and batch dataset submission from local computers or servers that could be connected. Up to now, the supported software and device are listed in Table 1. Standardized data submission modules can not only collect source data files but also parse data from source data, facilitating the application of standardized data. Fig. 4 shows an example of a standardized data application. Data extracted from the VASP calculation files, including INCAR, KPOITS, OUTCAR, and vasprun.xml, can be visualized by matcloud^3^, displaying the Lattice structure and parameters. Though we only parse the meta data of ‘results files’ calculated from VASP, ABINIT and LAMMPS software. (*e.g*., simulation input parameters, software version, results information). For ‘results files’ generated by other software, we simply store them as-is. They can be parsed by special software, and the analyzed results can again be stored in NMDMS using our third-party interfaces.

### Data publication

**Fig. 5:**
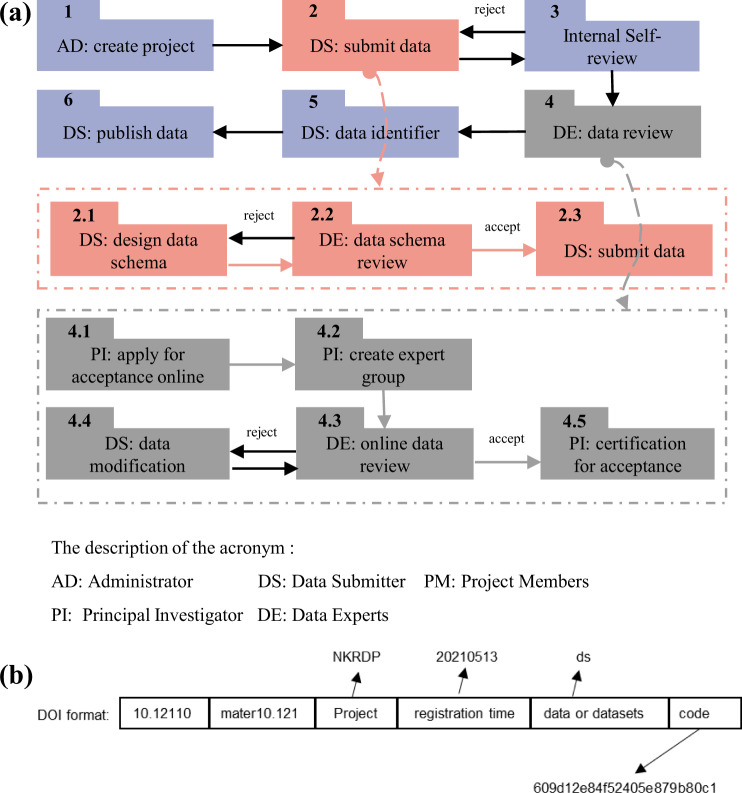
A rigorous project scientific data review and publication process. (**a**) Flow chart of data submission, review, acceptance, and publication. The red dotted box represents the data submission process. The gray dotted box represents the flow chart of the data submission and acceptance process. (**b**) The format of data digital object identifier (DOI) in NMDMS.

A personalized and Standardized Material Data Submission system can accelerate the collection of multi-domain material data, but it is equally crucial to ensure the data be traceable and of high quality. Traceable data is the basis of counting and evaluating project data. From the aspect of data quality, although part of the calculation and experimental data are standardized by using the high-throughput calculation data submission module and experiment device data submission module, most data are still personalized, and the data quality is uncontrollable, making it difficult to reuse. Therefore, as Fig. 5 shows, to trace the data status and control data quality, we design a concise and efficient online data submission, review, and acceptance process, similar to the paper publishing process.

1. **Create Project**. Project and user information is essential to associating submitted data with specific projects and users. Hence, a project is created by the administrator before data submission, and a principal investigator (PI) account is established for the project (Fig. 5a.1). The PI can edit and submit the project information online, including project name, ID, members, and data evaluation indicators. In practice, the PI is assigned by the project management department, which can always be trusted. The PI should know well about the domain experts, who are familiar with the data generated by the project. In step 4 of the data review process (Fig. 5a.4), the project-defined evaluation indicators will be used as the reference indicators to evaluate the project data by external data experts.
2. **Submit Data**. Before submitting data, project members need to choose or design data schemas. To ensure data quality, data experts (DE) are assigned by PIs in various materials fields. DE’s authority can be ensured via this chain of trust. Also, this process is carried out within the project team, and they can help us continuously obtain more data experts in various materials fields. And the expertise and appropriateness of domain experts eventually improves through this virtuous circle. If the data schema is rejected, the data schema needs to be revised. Only approved data schema could be used to submit data via the Personalized and Standardized Material Data Submission system (Fig. 5a.2).
3. **Internal Self-review**. After submitting the data, the PI needs to assign project members to review the details of the data to assess its quality and decide whether it should be approved. Only approved data can be included in the follow-up data review list. For the unaccepted data, the data submitter can withdraw and resubmit the data after modifying it.
4. **Data Review by Experts**. As Fig. 5a.4 shows, PI can apply for the data acceptance assessment once all project data has been submitted and internally reviewed. Then, an external expert group will be assigned to review and evaluate the project data quality to determine whether the project data can be accepted. The project team can modify the data based on the comments of data experts. After all of the experts accept the project data, the PI can view and download the project data acceptance report, which is one of the most critical evaluation metrics of project performance.
5. **Data Publication**. The accepted data will be published in NMDMS with an internal data unique identifier. NMDMS also provides the registration of a digital object identifier (DOI) for dataset records. Anyone who uses the data from NMDMS needs to cite data in the format shown in Fig. 5b.

### Data sharing

The above two systems assure that data is interoperable, reusable, and accessible. The query method of data can effectively facilitate the finding and sharing of data. To retrieve data for different application scenarios, NMDMS provides three types of query methods: Full-text, SQL-like query, and knowledge graph-based query.

**Fig. 6:**
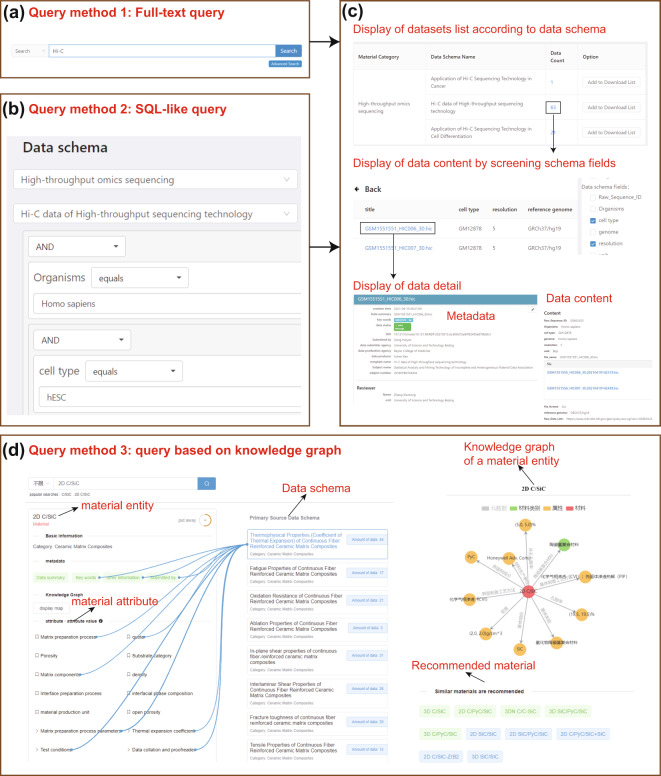
A multi-granularity data query and acquire subsystem. (**a**) the interface of “keyword query”. (**b**) the interface of “SQL-like” query. (**c**) the interface of three types of data display. (**d**) the interface of “query based on knowledge graph”.

Full-text query and SQL-like query (Fig. 6a,b) are both developed based on the powerful index of ‘Elasticsearch^50^’. The metadata and dataset content are stored based on the semi-structured dynamic container database. Therefore, the metadata and dataset content can be indexed in Elasticsearch, enabling a query of the dataset content. As shown in Fig. 6c, the query result of the above two methods is displayed in three forms. Firstly, the datasets are listed with material category, data schema name, data count, and download buttons. Secondly, by clicking on the “data count” of the dataset list, all the data content is listed as a table. The table form can provide a global overview of the dataset. The content of the table can be user-defined by selecting the fields of the data schema. Lastly, a document containing all information about the data metadata, data schema, and data content is displayed by clicking on the title of the data list.

SQL-like query based on the attributes of data schema provides a ‘SELECT’ -like function to query data belonging to a specific data schema. As Table 2 shows, the ‘SELECT’ -like function defines 22 operators, including arithmetic operators, quantifier operators, and logic operators for conducting the query criteria of the dynamic container data content. For example, to find the 2D SiC/PyC/SiC material datasets belonging to the data schema ‘Fatigue Properties of Continuous Fiber Reinforced Ceramic Matrix Composites, the following query criteria can be used (Fig. 6b): ‘grade’ equals ‘2D SiC/PyC/SiC’ AND ‘interfacial phase composition’ equals to ‘PyC’. Users who know the data schema structure are more likely to benefit from a SQL-like query.

**Table 2:** The definition of operations of the SQL-like query.

Operator type	Operator name	object	note
Arithmetic operator	null	all	is null
	any		is not null
	eq	String, Number	be equal to …
	neq		not be equal to …
	start_with	String, Choice	prefix with …
	nstarts_with		not prefix with …
	ends_with		suffix with …
	nends_with		not suffix with …
	contains		contain string …
	ncontains		not contain string …
	eq	Number	be equal to …
	neq		not be equal to …
	gt		more than …
	lte		less than or equal to …
	lt		less than …
	gte		more than or equal to …
Quantifier operator	all satisfy	Table, Array	all rows that satisfy …
	exist none that satisfies		There is no row exists that satisfies …
	exist one that satisfies		There is a row exists that satisfies…
	exist k-row that satisfies		There is k-row exists that satisfies…
Logic operator	and	—	When the query is satisfied at the same time, the query result is returned
	or		When one of the queries is satisfied, the query result is returned

NMDMS may have several datasets with different data schemas that describe the same material due to the personalized data submission. However, the above two query methods cannot simultaneously display all the data content from multiple data schemas. Datasets are reorganized as a knowledge graph automatically by leveraging the tree structure of data schema. Therefore, as shown in Fig. 6d, we also provide a query method based on the knowledge graph (KG-based query) to display the combination of multiple datasets with different data schemas. For example, as Fig. 6d shows, the ‘2D C/SiC’ query results include a material entity, metadata, attributes, and knowledge graph merged from eight data datasets. The blue lines describe the affiliation of data attributes to the data schema. Similar materials are recommended to help researchers find more materials with a similar attribute value.

### Application

**Fig. 7:**
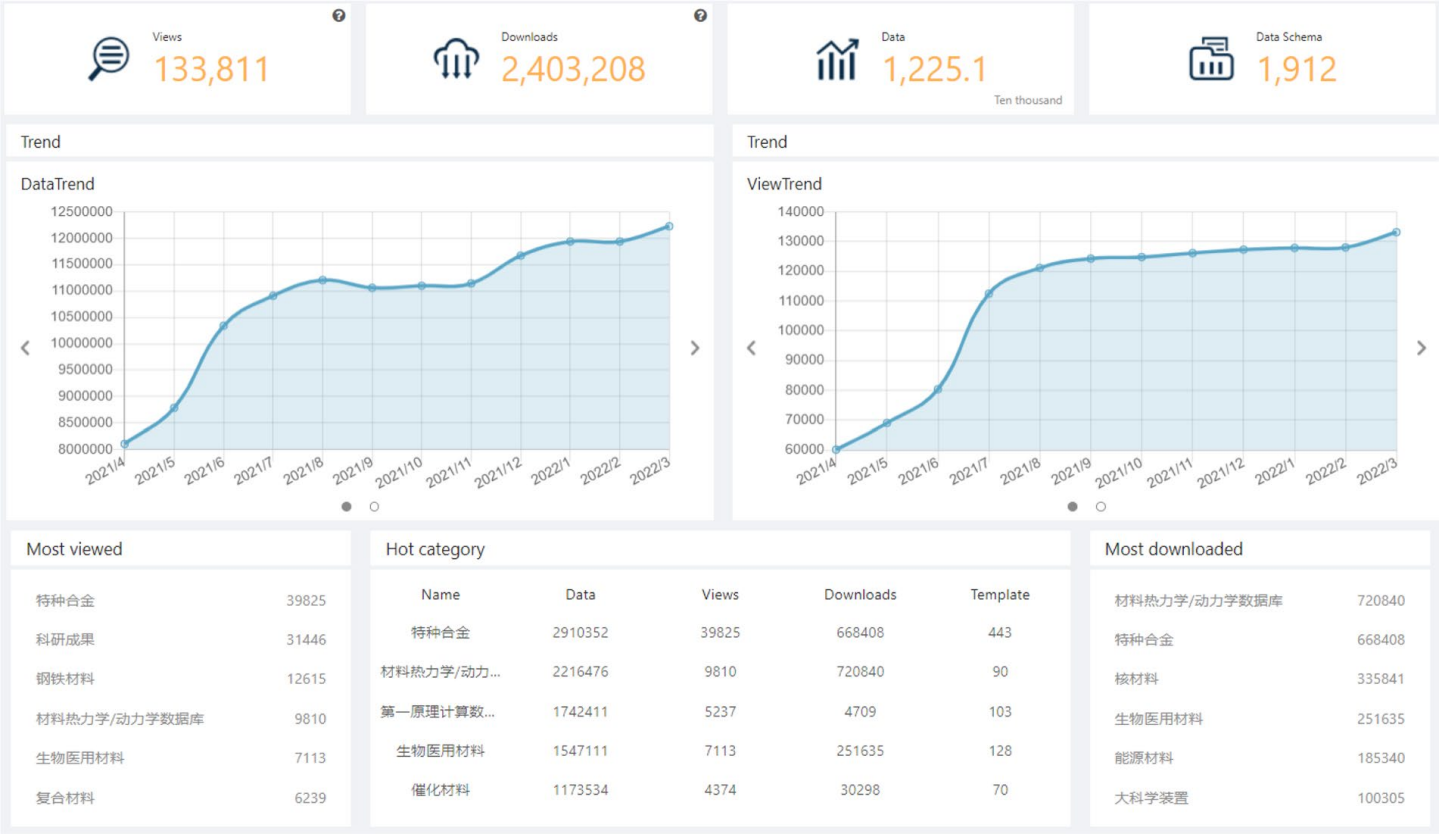
The statistics interface of NMDMS. The statistics information of data submissions, data schema submissions, data view, and data downloads is shown in a line chart. The bottom of the interface shows the hot data categories that are most viewed, and most downloaded.

Since 2018, NMDMS has been used for national materials genome engineering data publication and sharing. NMDMS has registered 1610 users and 45 projects. As Fig. 7 shows, these users have designed 1,912 data schemas and published 12,251,040 data records scattered in 87 material categories. The number of platform visits is 908875. The data view is 133,811, and the data downloads are 2403,208. NMDMS contributes to material data sharing and repurposing to accelerate the research and development of new materials. Based on the evaluation report of submission data, 36 projects have completed the project performance evaluation. To help users perform machine learning using the retrieved data from NMDMS, data in NMDMS, data can be visually displayed in two-dimensional tables via reorganizing and transforming. Meanwhile, the retrieved data can be exported by field in various formats (as shown in Supplementary Figure S1), which is conducive to the reusability of machine learning tools. By calling the data exporting interface, third-party tools can use the selected data fields as inputs of other machine learning tools. More machine learning tools can also be integrated into NMDMS through the interfaces. Therefore, with various ways of data retrieval, export and transforming, NMDMS facilitates the discovery of knowledge.

## Discussion

The dynamic container technology^45^ aims to solve the problem of inefficient storage and retrieving of heterogeneous material scientific data, and enables users to define personalized data schema and upload user-defined data. By leveraging the dynamic container database’s superiority in storing heterogeneous data, we built the material scientific data publication and sharing platform supporting the FAIR principle and collected 12,251,040 data records from 87 material categories of the 1912 data schema. To help researchers use NMDMS to do scientific discovery, NMDMS provides the technology supporting multimodal data storage and query. NMDMS also provides abundant interfaces to communicate with third-party tools, and we are trying our best to optimize these interfaces for easier usages. Here we describe the two viewpoints in details.

MDCS is a platform to submit and query user-defined data sharing a similar idea to NMDMS. Here, we describe the advantages of NMDMS compared with MDCS (Table 3) to construct the material data publication and sharing platform.

**Table 3:** The comparison of dynamic container technology and MDCS from the aspect of data type, create data schema, submit data query data and export data.

Functions	MDCS	NMDMS:
		Dynamic Container Technology
Data type	35 data types	**10 streamlined data types**
	Table and array type are not supported	Table and array type are supported
Create schema	XML tree	**Directory tree**
	a single layer can be displayed when creating a data schema	**Complete data schema is displayed**
	Click to build data schema	**Drag** to build data schema
Submit data	Personalized data submission	**Personalized and standardized data submission**
	Personal data publication	**Project data publication with a rigorous data review process**
Query data	Key words to query	Key words to query
	SQL-like query	SQL-like query
	—	**Query data across schema based on knowledge graph**
Export data	API	API
	XML format	**EXCEL, JSON** formats

Firstly, in contrast to MDCS, which offers thirty-five types of data attributes, dynamic container technology provides ten data attributes that are much easier to comprehend for non-computer professionals.

Secondly, NMDMS provides a much more user-friendly human-machine interface (HMI) to create data schemas. As shown in the video of creating a data schema (https://www.youtube.com/watch?v=G0_54ST97Ns), the operation of dragging and dropping components to create a data schema is fast, efficient, and straightforward. The display of data schema by using a directory tree is intuitive. However, MDCS only shows the XML tree structure of the data when creating the data schema, leading that we can only know whether the data structure is correct or not when the data is submitted.

Thirdly, from the aspect of data submission and publication, MDCS only provides a personalized data submission module to publish data. NMDMS provides four data submission modules to realize the personalized and standardized project data submission, a rigorous data submitting, reviewing, and publication process to ensure data quality.

Fourthly, as for data querying, in addition to the keywords query and SQL-like query, NMDMS also provides a data query method based on a knowledge graph to query data combined from different data schemas.

Lastly, NMDMS provides the export data function supporting EXCEL and JSON data formats, where the EXCEL format is much easier to be understood for non-computer professionals.

Based on the description of the FAIR principle (Supplementary Table S1), NMDMS satisfies the four principles as follows. (1) Findable: F1. Data in NMDMS are assigned with an internal data unique identifier. NMDMS provides the registration of a digital object identifier (DOI) for dataset records. F2. 1) Data content in NMDMS can be described using rich metadata using the function of Data Schema Designer. 2) Metadata fields used to describe the basic information about a dataset is set in NMDMS. F3. metadata in NMDMS clearly and explicitly include the identifier of the data it describes. F4. data are registered using DOI and other data fields are indexed and searchable using elastic search. (2) Accessible: A1. Data in NMDMS are retrievable by DOI, title, author, and any data content included in the data. A2. Metadata in NMDMS is stored independently. Hence, metadata can be searched, even when the data are no longer available. (3) Interoperable: I1. Metadata, data schema, and data content can be represented in English or any other language for knowledge representation. I2. Data schema integrates the Materials lexicon generated by the book of materials comprehensive dictionary. This follows FAIR principles. I3. Metadata in NMDMS provides the reference field to describe the source data or the other data related to the data. (4) Reusable: Data in the NMDMS platform can be described with as many attributes as possible selected from the Materials lexicon using the function of Data Schema Designer.

In conclusion, compared with the MDCS, NMDMS provides a more user-friendly interface based on the dynamic container technology to publish and share data. What’s more, the application of NMDMS for the national materials genome engineering data publication and sharing illustrates the practicality of dynamic container technology. In the future, NMDMS will aim to explore data quality analysis algorithms to filter the high-quality dataset and develop a series of tools to do data mining.

## Methods

### Architecture

NMDMS adopts a browser-server architecture based on the dynamic container technology^45^ that allows users to easily publish and share data on the platform through a browser. NMDMS is currently online and accessible at http://nmdms.ustb.edu.cn.

**Fig. 8:**
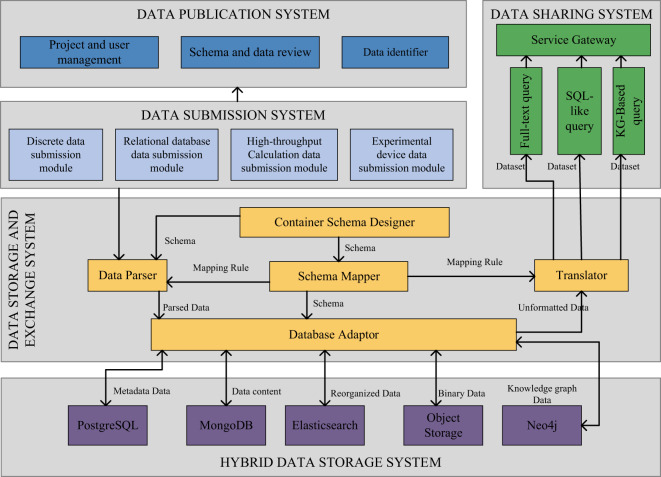
The architecture of NMDMS consists of five systems: HDSS, DSES, DSuS, DPS, and DShS. HDSS stores each category of data in the corresponding database. DSES is responsible for data schema creation and data parsing. DSus is responsible for submitting multiple data types of data. DPS manages the project information and data evaluation process. DShS provides three data querying methods for sharing data.

Fig. 8 provides an overview of NMDMS’s architecture. NMDMS has five main systems, including hybrid data storage system (HDSS), data storage and exchange system (DSES), data submission system (DSuS), data publication system (DPS), and data sharing system (DShS). Details of HDSS and DSES have been described in MGED^45^. DSuS provides discrete data, relational database data, high-throughput calculation data, and experiment device data submission modules to collect personalized and standardized data. PMS manages the project information and the data evaluation process to publish data. DShS provides three data querying methods, including Full-text query, SQL-like query, and KG-based query.

### Definition, exchange, and storage of scientific data based on dynamic container

#### Structure and storage of scientific data based on dynamic container

**Fig. 9:**
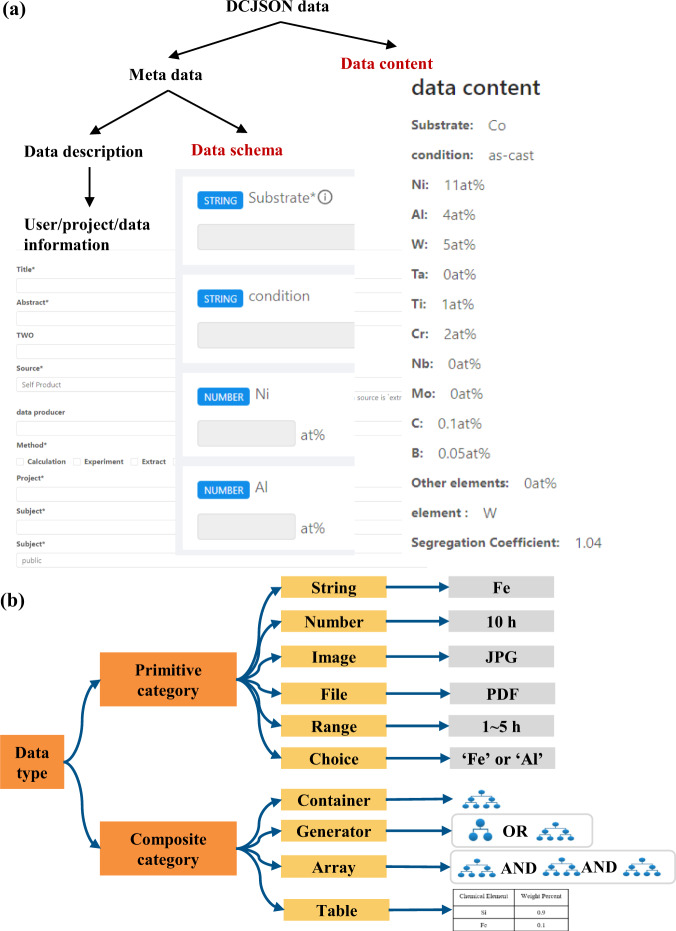
The description of data stored on the dynamic container. (**a**) The structure of DCJSON data. (**b**) The description of ten data types.

As Fig. 9 shows, data submitted based on the dynamic container technology is stored as a JSON document in a special format called dynamic container JSON (DCJSON). DCJSON consists of metadata (i.e., data description, data schema) and data content.

JSON data can be combined to be any format with the six basic data types, including string, number, null, objective, array, and bool. For the parts of data schema and data content, the DCJSON defines 10 data types based on the six data types of JSON document (Table 4) to limit the JSON document structure, which is much easier to be understood to store the material data. As Fig. 9b shows, the ten data types can be classified into two categories: 1) The primitive data types, including string, number, image, file, range, and choice aim to define the data attributes. 2) The composite data types, including array, generator, container, and table, aims to construct the organization of the data structure. The container is a nested data type containing any data types. The array type of data represents a group of data with a homogeneous data type with various numbers of elements. The generator represents a data type that can be selected from a group of heterogeneous data types when submitting data.

**Table 4:** The data types of the JSON and DCSJON document.

Data Category	JSON document	DCJSON document
primitive	String, Number, Boolean, null	String, number, image, file, range, choice
composite	Object (JSON object), Array	Container, Array, Generator, Table

For each data format (Excel, JSON, CSV, XML), a schema file (or a copperplate file) will be automatically generated for users to fill in the data. The file contains data description (metadata), and data content (Supplementary Figure S2). The file structure may vary according to the file format, and users only need to fill in the value of data. They don’t need to organize the data themselves. The process of parsing the file into DCJSON document format consists of three steps: 1) Read metadata, of which the fields are specified by NMDMS rather than the user-defined schema; 2) Read the user-defined schema to parse the type of each schema field; 3) Read the data content, mapping each value to the corresponding field in the schema. The schema mapper iteratively reads each field in the file.

For the discrete data submission module, users can transform their raw data into the schema file (filling the schema file) manually or using a data format conversion program; The relational database data module automatically transforms relational data into schema files by mapping the data fields created by the data schema designer; The high-throughput calculation data submission module firstly transforms and processes the VASP, ABINIT and LAMMPS file into extracted data (e.g., simulation input parameters, software version, results in information), then transforms the extracted data into schema files; The experiment device data submission module defines the standardized data schema and transmits data between the experiment device data submission module and dynamic container module by using the schema file.

#### Definition of data schema and its operator

Definition of the data type declaration expression: If the type of the attribute *X* is *T*, then the data type declaration expression is defined as *X*:*T*.

Definition of data schema fragment: A data schema fragment *SF*_*j*_ is defined as a set of data type declaration expressions $S{F}_{j}={\left\{{X}_{i.j}:{T}_{i,j}\right\}}^{i\in 1,...,n}=\left\{{X}_{1.j}:{T}_{1,j},{X}_{2,j}:{T}_{2,j},...,{X}_{n,j}:{T}_{n,j}\right\}$, where n is the number of attributes.

Definition of data schema: A data schema *S* is defined as a set of data type declaration expression with a unique schema ID *SID*, $S={\left\{SID:{X}_{i}:{T}_{i}\right\}}^{i\in 1,...,n}=\left\{SID:{X}_{1}:{T}_{1},SID:{X}_{2}:{T}_{2},...,SID:{X}_{n}:{T}_{n}\right\}$, where n is the number of attributes. A data schema *S* can be a combination of multiple data schema fragments, i.e., $S={\left\{SID:{X}_{i}:{T}_{i}\right\}}^{i\in 1,...,m}\cup S{F}_{1}\cup S{F}_{2}...\cup S{F}_{n}$, where m is the number of attributes defined in *S*, n is the number of data schema fragments.

Definition of the data schema operators: v The creation operator of data schema is defined as *CreateSchema*(*S*), where *S* is the data schema *S*. The appending operator of data schema is defined as $AppendSchema\left(S,{S}_{append}\right)=S\cup {S}_{append}$. The updating operator of data schema is defined as $UpdateSchema\left(S,{S}_{new}\right)={\left\{SID:{X}_{i,new}:{T}_{i}\right\}}^{i\in 1,...,n}$. The deleting operator of data schema is defined as $DeleteSchema\left(S,{S}_{del}\right)=S-{S}_{del}$. The export operator of data schema is defined as *ExportData*(*SID*), where $SID={\left\{SI{D}_{i}\right\}}^{i\in 1,...,n}=\left\{SI{D}_{1},SI{D}_{2},...,SI{D}_{n}\right\}$, n is the amount of data schema to export. *ExportData*(*SID*) returns a set of data schemas *S* according to the set of data schema ID *SID*.

#### Definition of dataset and its operator

Definition of the assignment of attributes: If an attribute x has a value v, then the assignment expression is defined as x = v.

Definition of the data description: A data description M is defined as a set of fixed field F and corresponding value V, which can be expressed as $M={\left\{{F}_{i}={V}_{i}\right\}}^{i\in 1,...,m}=\left\{{F}_{1}={V}_{1},{F}_{2}={V}_{2},...,{F}_{m}={V}_{m}\right\}$, where m is the number of metadata fields.

Definition of the data content: A data content *C* is defined as a set of assignment expressions, which can be expressed as $C={\{{X}_{i}={v}_{i}\}}^{i\in 1,...,n}=\{{X}_{1}={v}_{1},{X}_{2}={v}_{2},...,{X}_{n}={v}_{n}\}$, where n is the number of attributes, *X*_*i*_ is the top-level attribute.

Definition of DCJSON data and dataset: A complete piece of data *D* is stored as DCSJON format consists of data ID, data description, data schema and data content. A piece of data j belonging to data schema i is defined as a pair (*DID*_*j*_, *M*_*j*_, *S*_*i*_, *C*_*j*_), where *DID*_*j*_ is the unique data id of data j. Then a dataset *DS* belonging to data schema i is defined as (*DID*, *M*, *S*_*i*_, *C*), where *DID* = {*DID*_*i*_}^*iϵ*1, …*n*^ = {*DID*_1_, *DID*_2_, …,*DID*_*n*_}, *M* = {*M*_*i*_}^*iϵ*1, …*n*^ = {*M*_1_, *M*_2_, …,*M*_*n*_}, *C* = {*C*_*i*_}^*iϵ*1, …*n*^ = {*C*_1_, *C*_2_, …,*C*_*n*_}, where n is the number of piece of data.

Definition of the data operator: The creation operator of data is defined as *CreateData*(*D*), where *D* is the data based on the data schema *i*. The modifying operator of data is defined as $$UpdateData(D,{D}_{new})=$$$${\{DID,{M}_{new},{S}_{i},{C}_{new}\}}^{i\in 1,...,n}$$. The deleting operator of data is defined as $$DeleteSchema(D,{D}_{del})=D-{D}_{del}$$. The export operator of data is defined as *ExportData*(*DID*), where *DID* = {*DID*_*i*_}^*iϵ*1, …*n*^ = {*DID*_1_, *DID*_2_, …,*DID*_*n*_}, n is the amount of data to export. *ExportData*(*DID*) returns a dataset *DS* according to the set of data ID *DID*.

#### Data retrieving methods

**Fig. 10:**
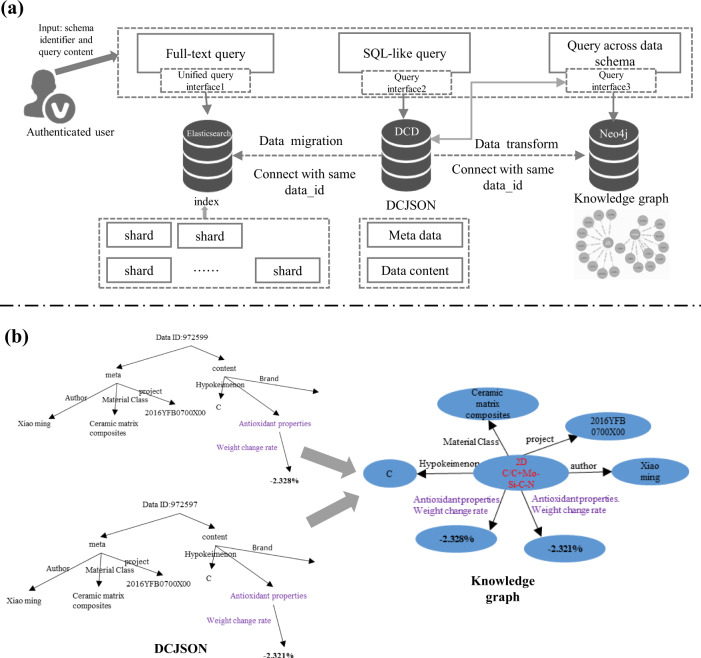
The description of three query methods. (**a**) Data retrieving methods including full-text query, SQL-like query and query data across data schema. (**b**) The generation of knowledge graph transformed from the data with DCJSON format.

As Fig. 10a shows, NMDMS realized the data retrieving method from three aspects: full-text query based on elastic search, SQL-like query based on schema fields and query based on the knowledge graph.

##### Full-text query

All DCJSON documents that contain meta data and data content are re-stored in Elasticsearch with index and document to realize the full-text query for metadata and data content. Since the DCJSON document is organized by the data schema, the Elasticsearch index is created with a unit of data schema. Then, functions such as full-text query and data filtering are realized through the Query DSL, word segmentation, relevance calculation and other functions from Elasticsearch.

##### SQL-like query

The top-level attribute is defined as the data field that could be described using the composite type. For example, a data schema S consisting of a top-level attribute *X*_*i*_, and *X*_*i*_ is described with a composite attribute *X*_*j*_. Then the path of data field *X*_*i*_ can be defined as ${X}_{{i}_{path}}=S.{X}_{j}.{X}_{i}$, the path of data content of data field *X*_*i*_ can be defined as ${D}_{{i}_{path}}=\left\{S.{X}_{j}.{X}_{i}={v}_{i}\right\}$. Based on the above definitions, similar to the operation of SQL querying, we define the Field Criteria and Criteria Group. (1) The Field Criteria is defined as ${C}_{f}= < Path,\,Operator,\,Value >$, where *Path* is the path of a data field, *Operator* is the arithmetic operators or quantifier operators defined in Table 3, *Value* is any values that the operator can accept. (2) Criteria Group: multiple Field Criteria can be combined using the logic operators (i.e., AND, OR). The combination is defined as *C*_*set*_ = <*Logic*, *S*_*c*_>, where *Logic* is a logic operator, *S*_*c*_ is a query set including a Field Criteria *C*_*f*_ or a combination *C*_*set*_. Since the data stored in dynamic container is realized as the JSON document, the Field Criteria is realized as the form of JSON document {“*path*”:*Path*, “*op*”:*Operator*, “*value*”:*Value*}, the Criteria Group is realized as the form of JSON document {“*AND*”:[*C*_*f1*_, *C*_*f2*_,…*C*_*fn*_]} or {“*OR*”:[*C*_*f1*_, *C*_*f2*_,…*C*_*fn*_]}, where ${C}_{i}\in {S}_{c}$, which could be represented by JSON values recursively. Based on the above definitions, the SQL-like query is developed to be a user-friendly human-machine interface (HMI) for users, instead of programing the query statement in a JSON format.

##### Query data across data schema based on the knowledge graph

Fig. 10b shows the automatic construction process of the knowledge graph based on the data with DCJSON format. By leveraging the heterogeneous data stored based on DCJSON, the knowledge graph is stored with a format <material entity, attribute, value>. The material entities are extracted from the data identifier (i.e., a field to describe data characteristics, such as material name, chemical formula, or grade) that must be defined in the data schema. The attributes are extracted by identifying the attributes of the data description and the path of a top-level attribute. The value is the data content assigned to be the attributes. In order to ensure the interpretability of the queried and recommended results, the attributes are all labeled with “material entity”, “material composition”, “structural feature”, “process preparation” or “experimental characterization methods”. The labels are assigned by calculating the semantic similarity between the path of top-level attribute and the terms come from “Materials Dictionary”, Wikipedia or Baidu Baike. Take material properties as an example. We classify and map 15 types of material properties, such as mechanics properties, physical properties, preparation process, chemical properties, electrical properties, magnetic properties, and so on. Based on the constructed knowledge graph, an interactive query and recommendation system was developed by calculating the similarity among the entity, label and value of these knowledge graphs.

## Supplementary information

Supplementary Information

## Data Availability

All data are stored in NMDMS (http://nmdms.ustb.edu.cn/). Anyone who register the platform can access these data by querying. The data query page (http://mged.nmdms.ustb.edu.cn/search/#/NMDMS) allows users who does not login in to query data. Any registered user can submit and download data on the platform. The description and usage of NMDMS can be seen from YouTube https://youtu.be/vhXIW9hGxwQ or the user manual file (Supplementary material M1).

## Code availability

The source codes contain various intellectual properties of our team members and we are not at liberty to distribute them. However, institutions may request for their own deployment of the system. Uers may contact the corresponding author (Jie He, email: hejie@ustb.edu.cn, Xiaotong Zhang, email: zxt@ies.ustb.edu.cn) for such requests.
