# Supplementary material for: A repository for the publication and sharing of heterogeneous materials data
Source: Sci Data. 2022 Dec 27;9:787. doi: 10.1038/s41597-022-01897-z (PMC9794830; doi:10.1038/s41597-022-01897-z)
Supplement: Supplementary file 1 — Supplementary Information [file 41597_2022_1897_MOESM1_ESM.pdf]

catalogue

Supplementary Table S1 2
Supplementary Table S2 3
Supplementary Table S3 4
Supplementary Figure S1 6
Supplementary Figure S2 6
References 7
Supplementary material M1 10
register and login 10
login 10
Register 10
Homepage 10
apply for permission 12
User manual for the discrete data submission module 14
Full-text query 15
SQL-like query 17
Data submission 17
Data schema creation 19
Analytics 20
Data schema review 20
Data review 21
Data certification 21
Register DOI 23
User manual for the relational database data submission module 24
User manual for the high-throughput calculation data submission module 24
User manual for the experiment device data submission module 25

Supplementary Table S1. Description of FAIR principle (cite from reference1)

FAIR principle	Description	how NMDMS satisfies
Findable	F1. (meta)data are assigned a globally unique and persistent identifier<br>F2. data are described with rich metadata (defined by R1 below)<br>F3. metadata clearly and explicitly include the identifier of the data it describes<br>F4. (meta)data are registered or indexed in a searchable resource	F1. Data in NMDMS are assigned an internal data unique identifier. NMDMS provides the registration of a digital object identifier (DOI) for dataset records.<br>F2. Data in NMDMS can be described using rich metadata using the function of Data Schema Designer.<br>F3. metadata in NMDMS clearly and explicitly include the identifier of the data it describes.<br>F4. (meta)data are registered using DOI and indexed using elastic search.
Accessible	A1. (meta)data are retrievable by their identifier using a standardized communications protocol<br>A1.1 the protocol is open, free, and universally implementable<br>A1.2 the protocol allows for an authentication and authorization procedure, where necessary<br>A2. metadata are accessible, even when the data are no longer available	A1. Data in NMDMS are retrievable by DOI, title, author and any data content included in the data.<br>A2. Data in NMDMS can be searched, even when the data are no longer available
Interoperable	I1. (meta)data use a formal, accessible, shared, and broadly applicable language for knowledge representation.<br>I2. (meta)data use vocabularies that follow FAIR principles<br>I3. (meta)data include qualified references to other (meta)data	I1. Metadata use English language for knowledge representation. Data schema and data content can be represented any other language.<br>I2. Data schema integrated the Materials lexicon generated by the book of materials comprehensive dictionary. This follows FAIR principles.
Reusable	R1. meta(data) are richly described with a plurality of accurate and relevant attributes<br>R1.1. (meta)data are released with a clear and accessible data usage license<br>R1.2. (meta)data are associated with detailed provenance<br>R1.3. (meta)data meet domain-relevant community standards	R1. Data in NMDMS platform can be described with as much as possible attributes selected from the Materials lexicon using the function of Data Schema Designer.

Supplementary Table S2. A list of specific materials database

Platform Name	Access link	
MPDS[2]: Materials Platform for Data Science	https://mpds.io/#start	Chemical data
ChemSpider[3]	http://www.chemspider.com/	Chemical data
PubChem[4]	https://pubchem.ncbi.nlm.nih.gov/	Chemical data
MatDB[5]		HTR materials data
NOMAD[6]	https://nomad-lab.eu/	computational
AFLOWLIB [7]	https://aflowlib.org/	computational
Harvard Clean Energy Project[8]	https://gist.github.com/jessiegt/5642460f061a39d1820e	computational
Materials Project[9]	http://www.materialsproject.org/	computational
open Quantum Materials Database[10]	https://oqmd.org/	computational
Inorganic Crystal Structure Database (ICSD) [11]	https://icsd.products.fiz-karlsruhe.de/	identified inorganic crystal structures
3D Materials Atlas[12]	https://gist.github.com/glahoti6/66b818c8c8bc539a9332	3D characterization
American Mineralogist Crystal Structure Database[13]	http://rruff.geo.arizona.edu/AMS/amcsd.php	Minerals
ASM Alloy Center Database[14]	https://www.asminternational.org/home/-/journal_content/56/10192/15468704/DATABASE	Alloys
ASM Phase Diagrams[15]	https://libraries.psu.edu/databases/psu01625	Thermodynamics
CALPHAD Data Informatics[16]	https://www.nist.gov/programs-projects/calphad-data-informatics	experimental and computational phase-based data
Cambridge Crystallographic Data Centre (CCDC)[17]	https://www.ccdc.cam.ac.uk/	Crystallography
CrystMet[18]	https://cds.dl.ac.uk/cgi-bin/news/disp?crystmet	Crystallography
Crystallography Open Database (CoD)[19]	http://www.crystallography.net/	Crystallography
Powder Diffraction File (PDF)[20]	http://www.icdd.com/products/index.htm	Crystallography
CatApp[21]	http://suncat.stanford.edu/theory/it-facilities	Catalysts

Supplementary Table S3. The comparison of FAIR principle satisfaction of general database.

Platform Name	Access link	Findable (aim for data content)	Accessible	Interoperable	Reusable
NMDMS	http://nmdms.ustb.edu.cn/	√	√	√	√
MGED[22]	https://mgedata.cn/	√	√	Data may be unformal	Data may do not meet domain-relevant community standards
Citrination[23]	https://citrination.com/	√	√	× (Data may be unformal)	× (Data may do not meet domain-relevant community standards)
Materials Commons[24]	https://materialscommons.org/	× (Data content is stored without imposing rigid structure or file format restrictions)	√	×	×
DSpace[25]	https://materialsdata.nist.gov/dspace/xmlui		√	×	×
Dryad[26]	https://datadryad.org/stash		√	×	×
Figshare[27]	https://figshare.com./		√	×	×
Mendeley Data[28]	https://data.mendeley.com/		√	×	×
Zenodo[29]	http://zenodo.org/		√	×	×
DataHub[30]	http://datahub		√	×	×
DANS	http://www.dans.knaw.nl/		√	×	×
EUDat[31]	https://eudat.eu/		√	×	×
Data Citation Index [32]	https://www.ands.org.au/online-services/research-data-australia/data-citation-index		√	×	×
DataCite Search[33,34]	https://search.datacite.org/		√	×	×
DataMed[35]	Website cannot be reached		√	×	×
Dataset Search[36]	https://datasetsearch.research.google.com/		√	×	×
Material	https://materialsdatafacility.org/		√	×	×

Data Facility[37]					
AIST [38]	https://www.aist.go.jp/aist_e/list/database/riodb/		√	×	×
MatWeb[39]	https://www.matweb.com/		√	×	×
Granta CES Selector[40]	http://www.grantadesign.com/products/ces		× (Non-free)	×	×
Knovel[41]	https://app.knovel.com/kn/data-search			×	×
MATDAT[42]	https://www.matdat.com/			×	×
NIST Standard Reference Data[43]	https://www.nist.gov/srd/srd-catalog			×	×
Pauling File[44]	http://paulingfile.com/			×	×
SpringerMaterials[45]	https://materials.springer.com/			×	×
Total Materia[46]	https://www.totalmateria.com/page.aspx?ID=Home&LN=CN			×	×

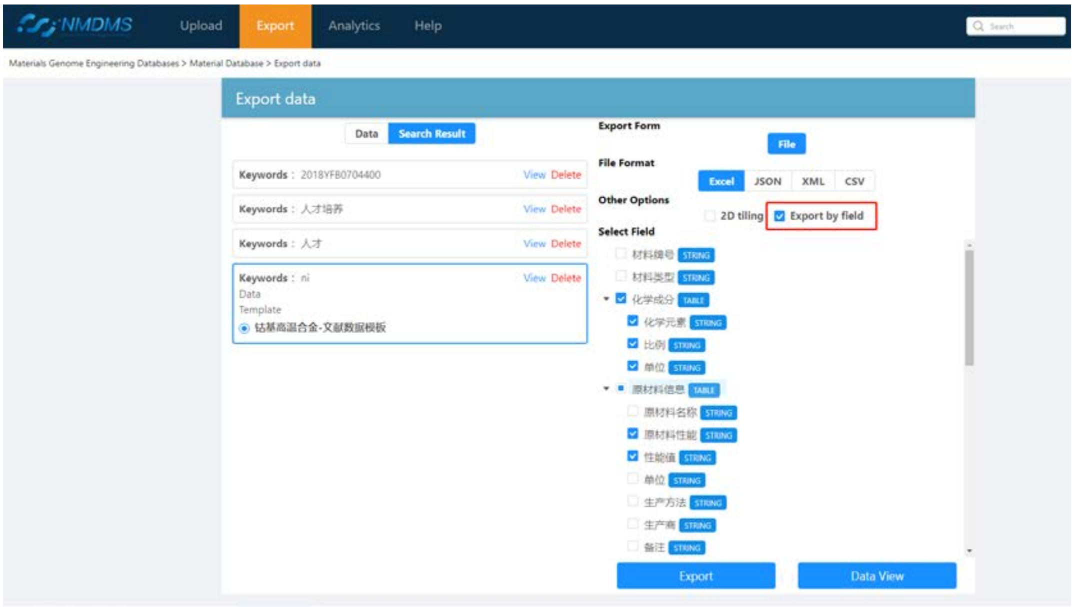

Supplementary Figure S1. The function and interface of exporting data by field.

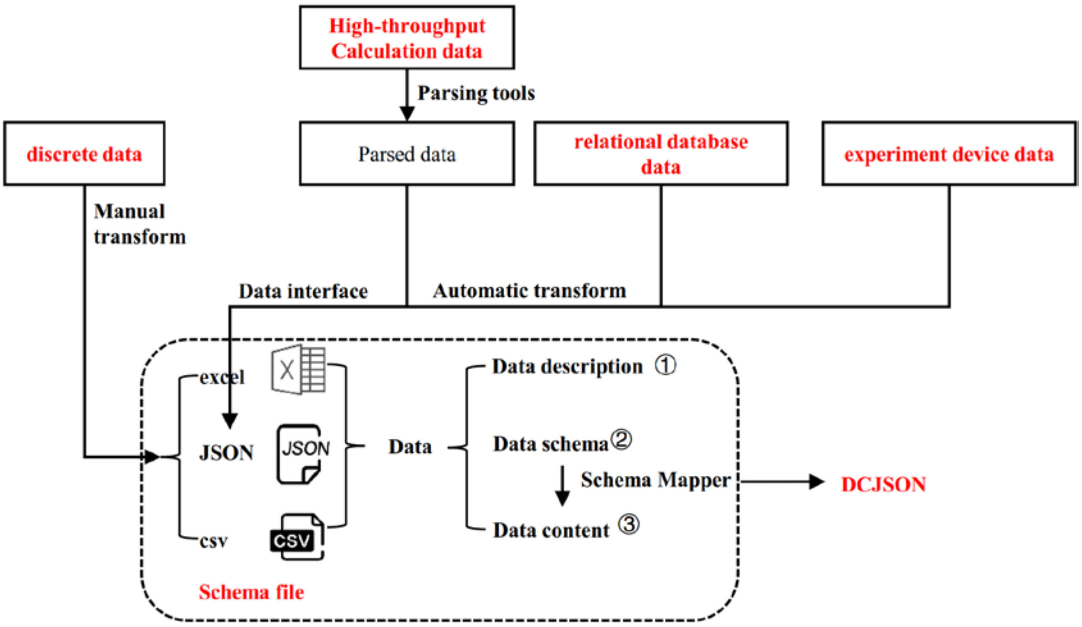

Supplementary Figure S2. The process of transforming source data (discrete data, high-throughput Calculation data, relational database data, experiment device data) in DSES.

References

1 Wilkinson, M. D. *et al.* The FAIR Guiding Principles for scientific data management and stewardship. *Scientific data* **3**, 1-9 (2016).

2 Blokhin, E. & Villars, P. The PAULING FILE project and materials platform for data science: From big data toward materials genome. *Handbook of Materials Modeling: Methods: Theory and Modeling*, 1837-1861 (2020).

3 Pence, H. E. & Williams, A. ChemSpider: an online chemical information resource. *Chm.Educ* **87**, 1123-1124, doi:https://doi.org/10.1021/ed100697w (2010).

4 Kim, S. *et al.* PubChem 2019 update: improved access to chemical data. *Nucleic acids research* **47**, D1102-D1109 (2019).

5 Over, H. & Hähner, P. MatDB Online analysis of HTR materials data from European R&D. *Nuclear engineering and design* **251**, 317-324 (2012).

6 Draxl, C. & Scheffler, M. The NOMAD laboratory: from data sharing to artificial intelligence. *Journal of Physics: Materials* **2**, 036001 (2019).

7 Curtarolo, S. *et al.* AFLOWLIB. ORG: A distributed materials properties repository from high-throughput ab initio calculations. *Computational Materials Science* **58**, 227-235 (2012).

8 Hachmann, J. *et al.* The Harvard clean energy project: large-scale computational screening and design of organic photovoltaics on the world community grid. *The Journal of Physical Chemistry Letters* **2**, 2241-2251 (2011).

9 Jain, A. *et al.* Commentary: The Materials Project: A materials genome approach to accelerating materials innovation. *APL materials* **1**, 011002 (2013).

10 Kirklin, S. *et al.* The Open Quantum Materials Database (OQMD): assessing the accuracy of DFT formation energies. *npj Computational Materials* **1**, 1-15 (2015).

11 Hellenbrandt, M. The inorganic crystal structure database (ICSD)—present and future. *Crystallography Reviews* **10**, 17-22 (2004).

12 *3D Materials Atlas*, <https://gist.github.com/glahoti6/66b818c8c8bc539a9332> (2022).

13 Downs, R. T. & Hall-Wallace, M. The American Mineralogist crystal structure database. *American Mineralogist* **88**, 247-250 (2003).

14 *ASM Alloy Center Database*, <https://www.asminternational.org/home/-/journal_content/56/10192/15468704/DATABASE> (

15 Vander Voort, G. F. *et al.* ASM handbook. *Metallography and microstructures* **9**, 44073-40002 (2004).

16 *CALPHAD Data Informatics*, <https://www.nist.gov/programs-projects/calphad-data-informatics> (

17 Groom C R, Bruno I J, Lightfoot M P, et al.. The Cambridge Structural Database: Acta Crystallographica Section B. B72, 171-179 (2016).

18 White, P. S., Rodgers, J. R. & Le Page, Y. CRYSTMET: a database of the structures and powder patterns of metals and intermetallics. *Acta Crystallographica Section B: Structural Science* **58**, 343-348 (2002).

19 Gražulis, S., Merkys, A. & Vaitkus, A. Crystallography open database (COD). *Handbook of Materials Modeling: Methods: Theory and Modeling*, 1863-1881 (2020).

20 Gates-Rector, S. & Blanton, T. The powder diffraction file: a quality materials characterization database. *Powder Diffraction* **34**, 352-360 (2019).

21 Hummelshøj, J. S., Abild-Pedersen, F., Studt, F., Bligaard, T. & Nørskov, J. K. CatApp: a web application for surface chemistry and heterogeneous catalysis. *Angewandte Chemie International Edition* **51**, 272-274 (2012).

22 Liu, S. *et al.* An infrastructure with user-centered presentation data model for integrated management of materials data and services. *npj Computational Materials* **7**, 1-8 (2021).

23 O'Mara, J., Meredig, B. & Michel, K. Materials Data Infrastructure: A Case Study of the Citrination Platform to Examine Data Import, Storage, and Access. *JOM* **68** (2016).

24 Puchala, B. *et al.* The materials commons: a collaboration platform and information repository for the global materials community. *JOM.* **68**, 2035-2044 (2016).

25 Smith, M. *et al.* DSpace: An open source dynamic digital repository. (2003).

26 Isard, M., Budiu, M., Yu, Y., Birrell, A. & Fetterly, D. Dryad: distributed data-parallel programs from sequential building blocks. in Proceedings of the 2nd ACM SIGOPS/EuroSys European Conference on Computer Systems.59-72 (2007).

27 Thelwall, M. & Kousha, K. Figshare: a universal repository for academic resource sharing? *Online Information Review* (2016).

28 Bhoi, N. K. Mendeley data repository as a platform for research data management. *Marching Beyond Libraries: Managerial Skills and Technological Competencies*, 481-487 (2018).

29 Dillen, M., Groom, Q., Agosti, D. & Nielsen, L. H. Zenodo, an Archive and Publishing Repository: A tale of two herbarium specimen pilot projects. *Biodiversity Information Science and Standards* (2019).

30 Bhardwaj A, Karger D, Subramanyam H, et al. Collaborative data analytics with DataHub. Proceedings of the VLDB Endowment International Conference on Very Large Data Bases. 8, 12 (2015).

31 Lecarpentier, D. *et al.* EUDAT: a new cross-disciplinary data infrastructure for science. *International Journal of Digital Curation* **8**, 279-287 (2013).

32 Pavlech, L. L. Data citation index. *Journal of the Medical Library Association: JMLA* **104**, 88 (2016).

33 Dudek, J., Mongeon, P. & Bergmans, J. DataCite as a Potential Source for Open Data Indicators. ISSI. 2037-2042 (2019).

34 Brase, J. Making data citeable: DataCite. *Opening Science*, 327-329 (2014).

35 Chen, X. *et al.* DataMed–an open source discovery index for finding biomedical datasets. *Journal of the American Medical Informatics Association* **25**, 300-308 (2018).

36 Noy, N. & Brickley, D. Facilitating the discovery of public datasets. *Google Res. Blogpost. Available online at: https://research. googleblog. com/2017/01/facilitating-discovery-of-public. html* (2017).

37 Foster, I. *et al.* in *Big Data and High Performance Computing* 117-132 (IOS Press, 2015).

38 IMAI, N., SAKURAMACHI, H., TERASHIMA, S., ITOH, S. & ANDO, A. Database on internet for geological survey of Japan geochemical reference samples. *Geostandards Newsletter* **20**, 161-164 (1996).

39 Gao, Z.-Y. & Liu, G.-Q. Recent progress of web-enable material database and a case study of NIMS and MatWeb. *Journal of Materials Engineering* **3**, 89-96 (2013).

40 Design, G. CES Selector. *Cambridge, UK: Material Universe. Zugriff unter https://www. grantadesign. com* (2018).

41 Smith, B. knovel: Engineering & Scientific Online References. *Reference Reviews* (2002).

42 *MATDATA*, <https://www.matdat.com/> (2022).

43 Lemmon, E., Bell, I. H., Huber, M. & McLinden, M. NIST Standard Reference Database 23: Reference Fluid Thermodynamic and Transport Properties-REFPROP, Version 10.0, National Institute of Standards and Technology. *Standard Reference Data Program, Gaithersburg* (2018).

44 Villars, P. *et al*. The pauling file. *Journal of Alloys and Compounds* **367**, 293-297 (2004).

45 *SpringerMaterials*, <https://materials.springer.com/> (2022).

46 *Total Materia*, <https://www.totalmateria.com/page.aspx?ID=Home&LN=CN> (2022).

Supplementary material M1

User manual for NMDMS platform

register and login

login

If you already have an account, you can login in by entering username(or email address),password and verification code.

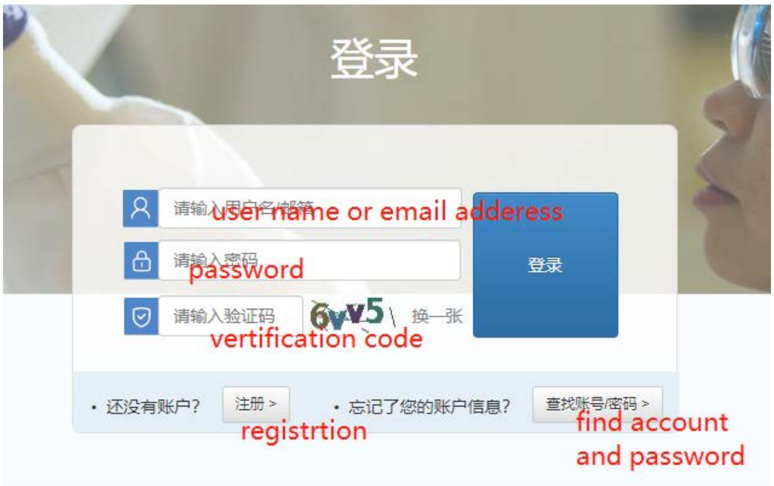

Register

If you do not have an account, you can click on “注册” to register an account by entering “username (or address), password, re-password, verification code, real name, institute and email address.

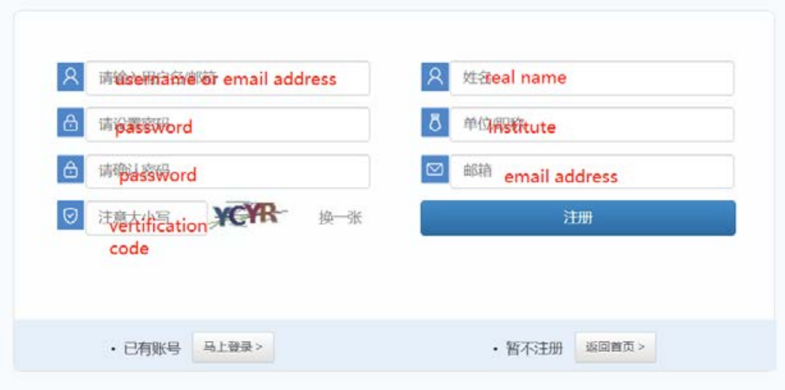

Homepage

After successful login, you could see the home page. By clicking on “离散数据汇交”, you can enter

the page of “the discrete data submission module”. By clicking on “高通量计算数据汇交”, you can enter the page of “The High-throughput calculation data submission module”. By clicking on “大科学装置实验数据汇交”, you can enter the page of “the experiment device data submission module”. By clicking on “关系型数据库数据汇交”, you can enter the page of “the relational database data submission module”. By clicking on “数据统计”, you can enter the page of “data statistics”. By clicking on “数据审核”, you can enter the page of “data review module”.

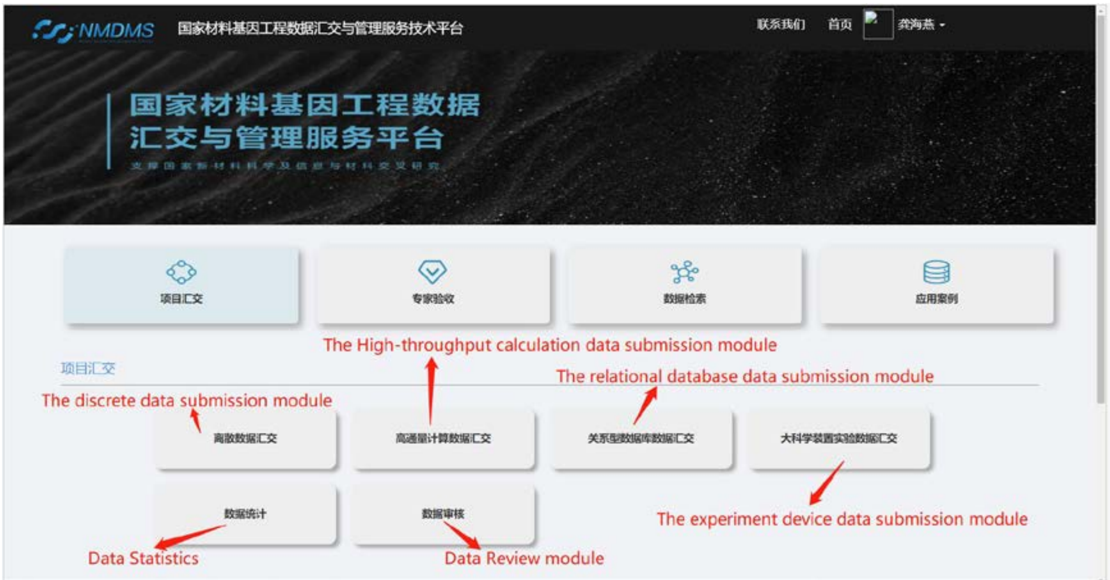

By clicking on “专家验收”, you can choose the following modules：项目信息管理（project information management system）、申请汇交证明（apply for data submission verification）、申请汇交验收（apply for data acceptance）、汇交验收分配（reviewer assignment）、汇交验收评价（data review by expert）.

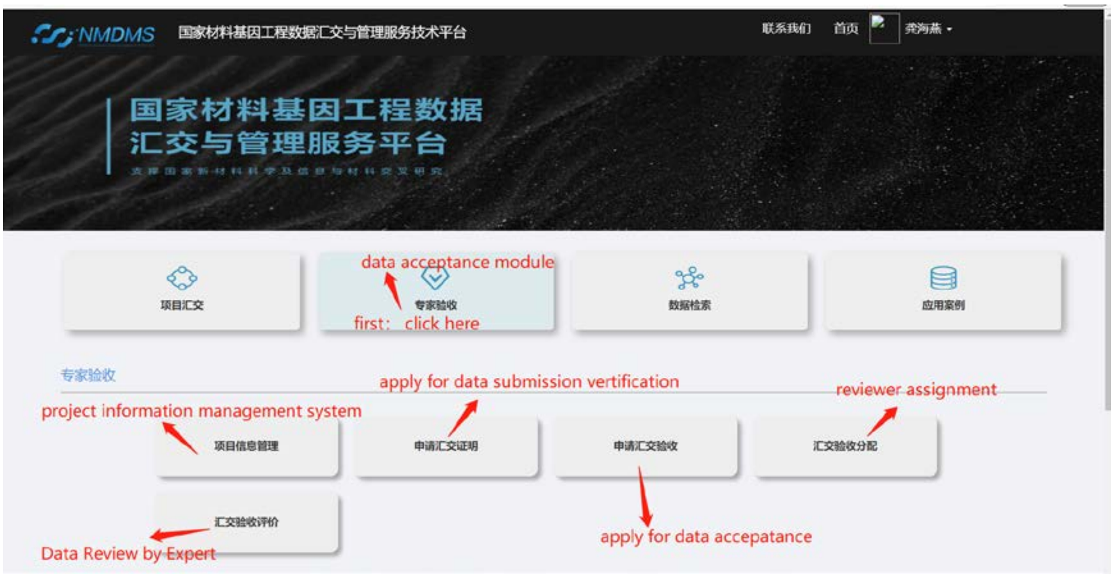

By clicking on “数据检索（Data retrieving system）”, you can choose the following modules：普通检索（Full-text query）、智能检索（query based on the knowledge graph）、高级检索（SQL-like query）.

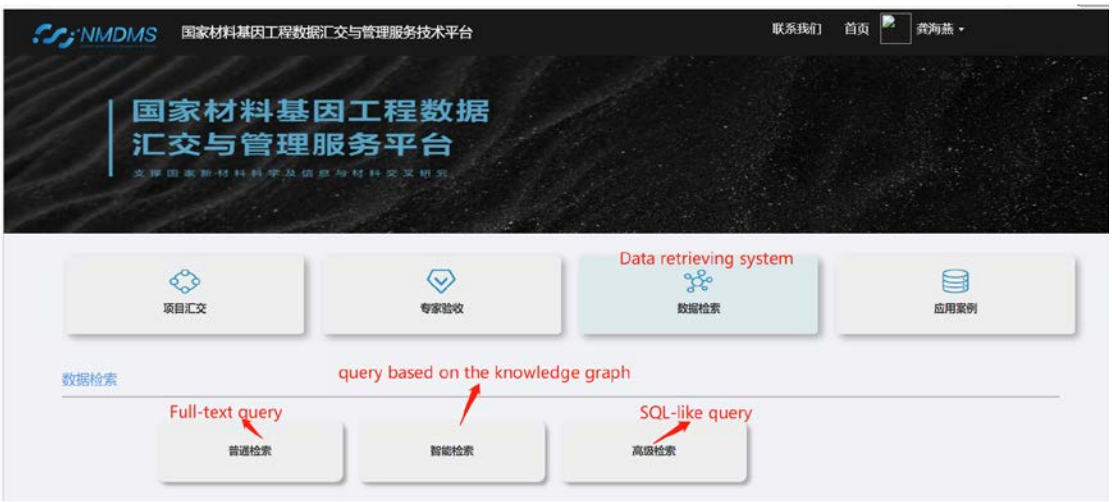

Due to the system's permission control Settings, only data retrieving system can be accessed without permission. For the submission module, we need to apply for permission.

apply for permission

click on the username, you can see the “个人信息（user information）”. Click on “个人信息”, and choose “角色权限（permission）”, and then click on “申请新权限（apply for new permission）”.

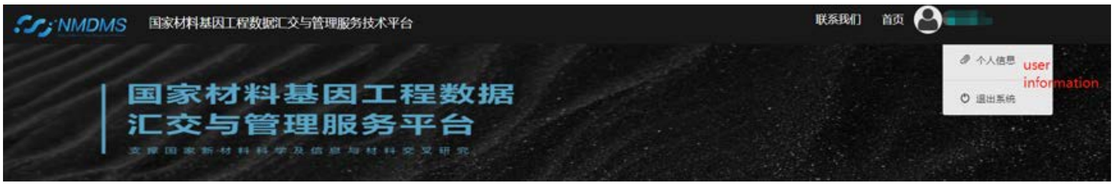

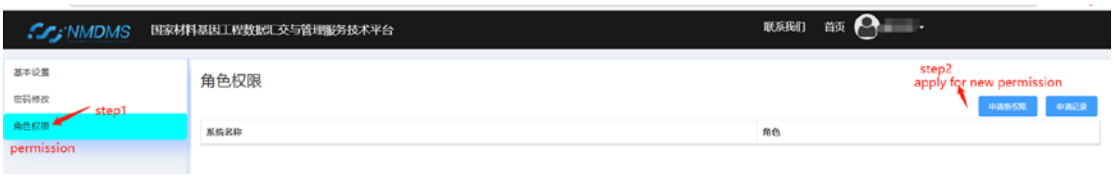

Choose “离散数据汇交（the discrete data submission module）”, and choose “数据上传（data submission）、“模板上传（schema submission）”. The other permission is not allowed for most users, such as “模板管理员（apply to be a reviewer for data schema）”,“数据管理员（apply to be a reviewer for data）”

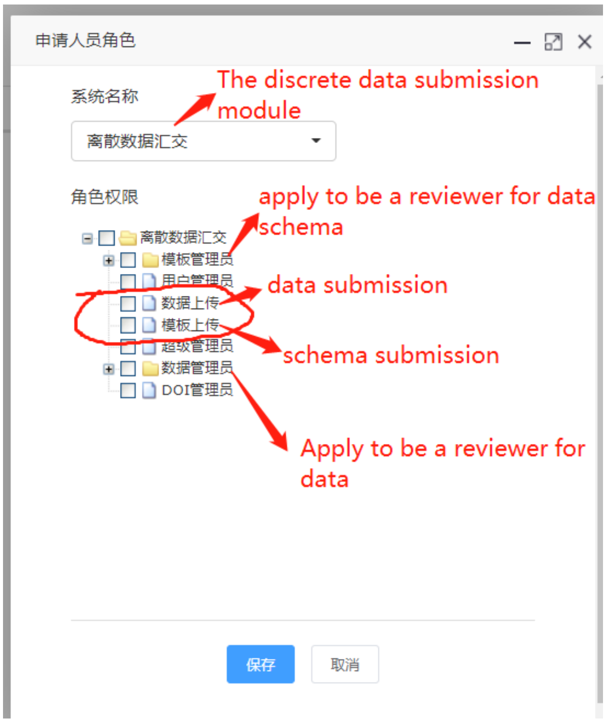

Choose “高通量计算数据汇交(the High-throughput calculation data submission module)”, and choose “算例上传（data submission）、“算例计算（calculation）”. The other permission is not allowed for most users, such as “管理员(Administrator)”.

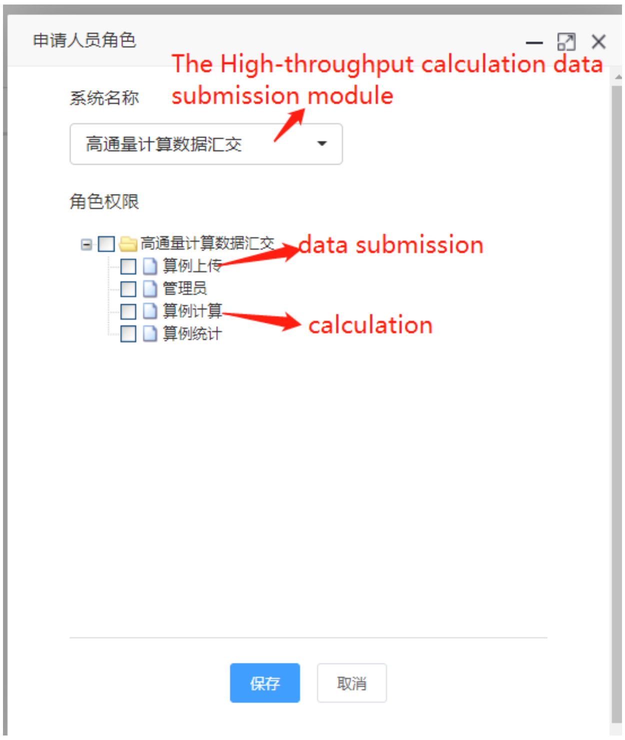

Choose “大科学装置实验数据汇交(the experiment device data submission module)”, and choose “普通用户（common user）”. The other permission is not allowed for most users, such as “管理员(Administrator)”.

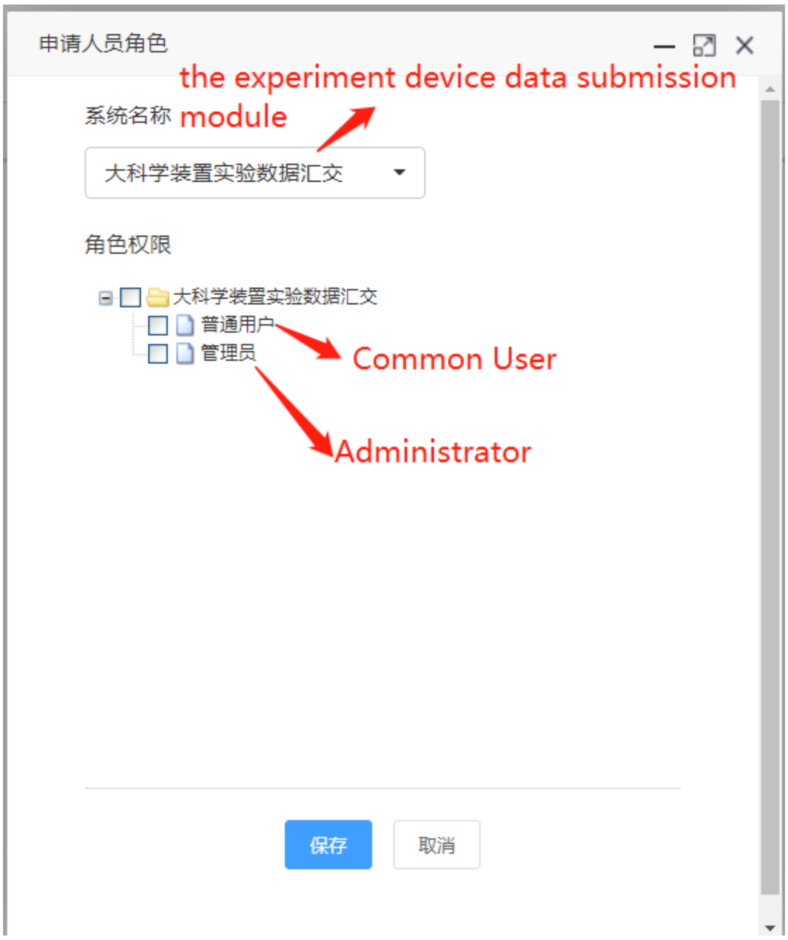

User manual for the discrete data submission module

The discrete data submission module includes data submission, data schema creation, data query and data review. If you just want to search for data, you can enter by login in or just click on the link http://mged.nmdms.ustb.edu.cn/search/#/. The discrete data submission module has two versions in Chinese and English. You can click on the button "En" to change this system to be an English version, or click on "中"to change this system to be a Chinese version. In the following manual, we will focus on the English version.

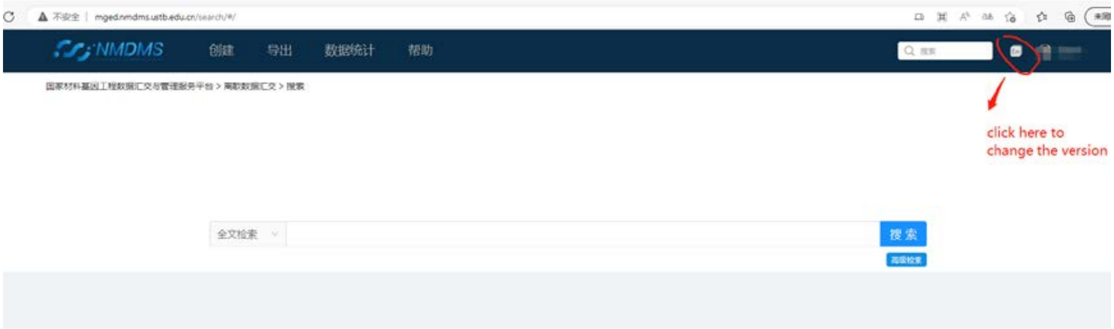

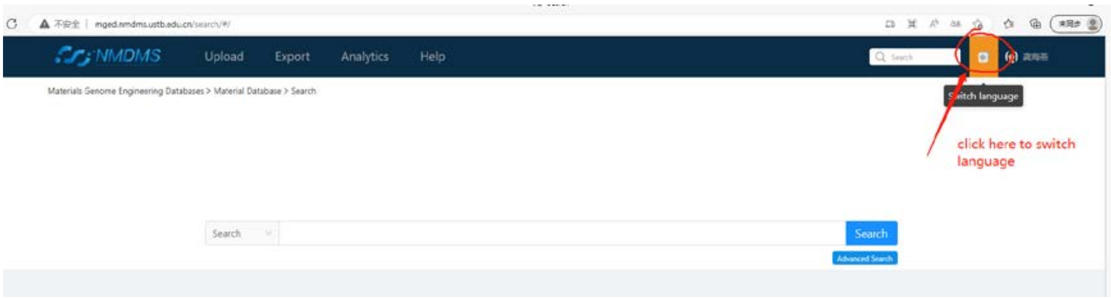

Full-text query

By click on http://mged.nmdms.ustb.edu.cn/search/#/, you can see the query box. You can enter the key words, such as Ni, datasets including Ni will be searched.

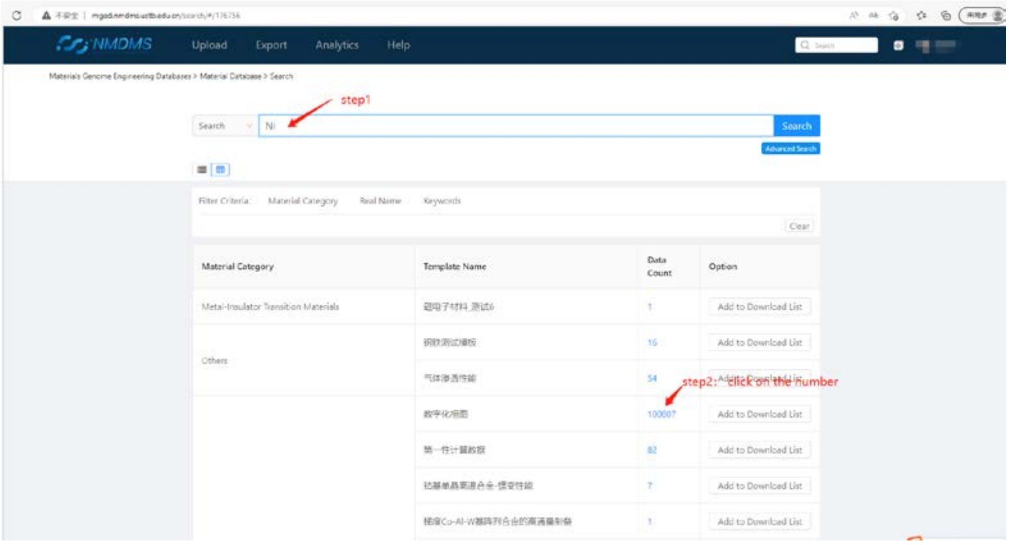

By Clicking on the bule number, you can the meta data information list. By screening on the fields right, you could choose the fields you want to see in the left table. By clicking on the title, you can see the data description.

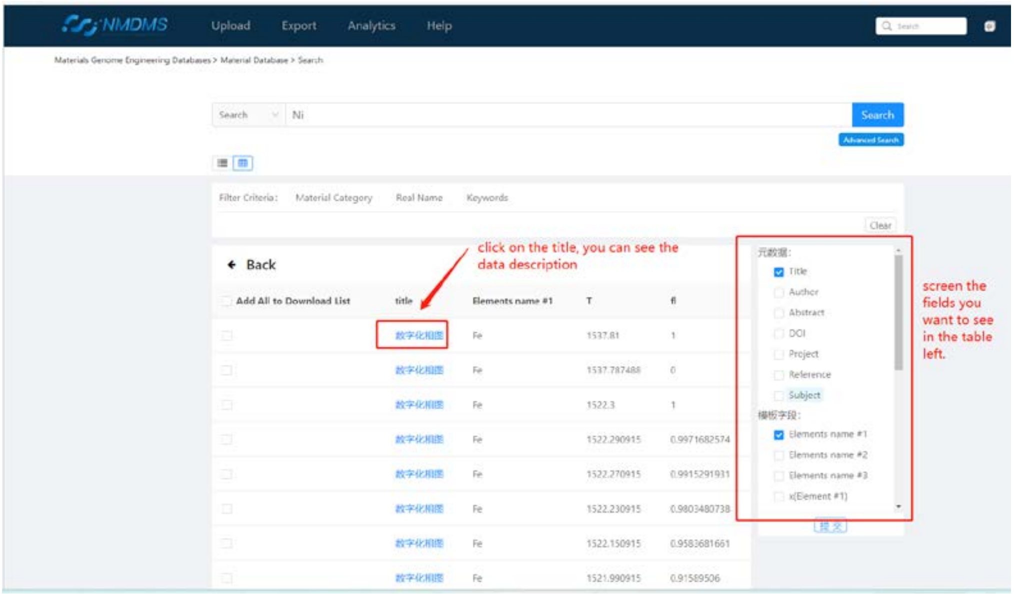

数字化相图

\+ Export 引 用

Created at 2022-09-01 13:34:26
Abstract 数字化相图
Keywords 数字化相图
Review state ✓ Approve
DOI DOI
Author 刘兴军
Data Contributor Institution 厦门大学
Responsible Institution 哈尔滨工业大学（深圳）
Contributor 龚晟楚
Template Name 数字化相图
Subject Name 材料大数据技术研究
Subject Number 2020YFB0704503

Reviewer

Real Name 潘登
Institution N/A

Content

Elements name #1: Fe
Elements name #2: Ni
Elements name #3: Co
x(Element #1): 1
x(Element #2): 0
x(Element #3): 0
T: 1537.81
fl: 1
fs: 0
Q: 0
H_Latent: 0
H_tot: 72465.22757
G: -108217.8205
f(@Liquid): 1
f(@Fcc): 0
f(@Fcc#1): 0
f(@Fcc#2): 0
f(@Bcc): 0
phase_name: Liquid+Bcc

Other Info

Source Self-production
Reference 引用
Method: computation,

Citations and Acknowledgments

To respect intellectual property rights, the protection of the rights of the data producer and provider, please users of the data based on the data generated by the research results (including the project report, academic thesis or dissertation, etc.) in a standard format for data in the reference and thanks:

Chinese reference: 国家材料基因工程数据汇交与管理服务平台.数字化相图。DOI:DOI.

English reference: National Materials Genome Engineering Data Collection and Management Service Platform.数字化相图。DOI:DOI.

Platform referenceLiu, S., Su, Y., Yin, H. et al. An infrastructure with user-centered presentation data model for integrated management of materials data and services. npj Comput Mater 7, 88 (2021). https://doi.org/10.1038/s41524-021-00557-x

Chinese acknowledgements感谢"国家新材料数据库平台建设关键技术研究"项目(2020YFB0704500)和国家材料基因工程数据汇交与管理服务平台（http://nmdms.ustb.edu.cn/）提供数据资源。

English acknowledgementsWe acknowledge for the data source from '国家新材料数据库平台建设关键技术研究'(2020YFB0704500) and "National Materials Genome Engineering Data Collection and Management Service Platform"(http://nmdms.ustb.edu.cn/).

2022 CopyrightCopyright©Materials Genome Engineering Databases
Online users: 1 Visits: 911641

SQL-like query

By clicking on the “advance search”, we enter the page of “SQL-like query”.

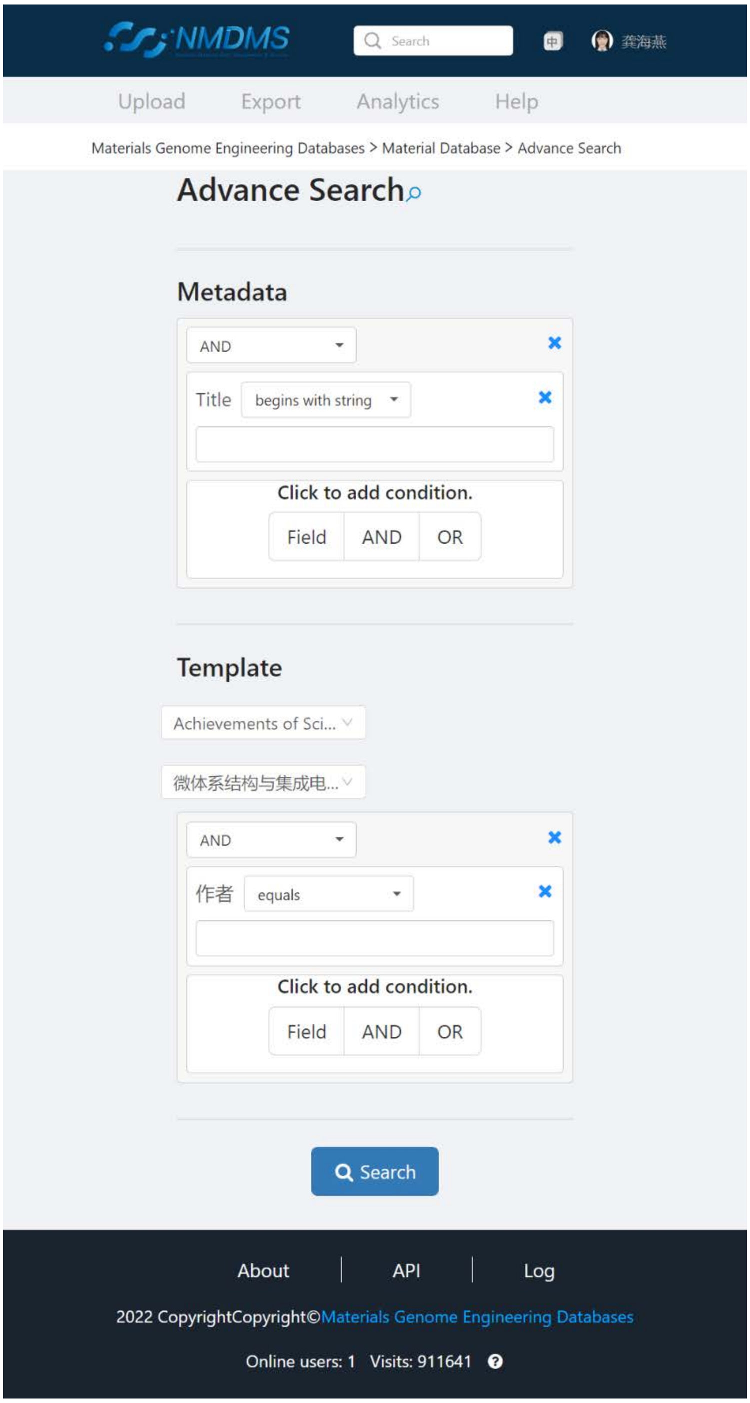

Data submission

By clicking on the Upload button, and choosing data, we can enter the page of data submission.

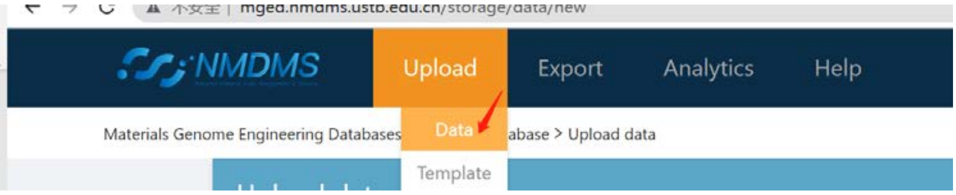

As shown below, the data includes two parts: meta data and data content. The meta data is defined by the NMDMS platform. The data content is generated by the data schema as users have created.

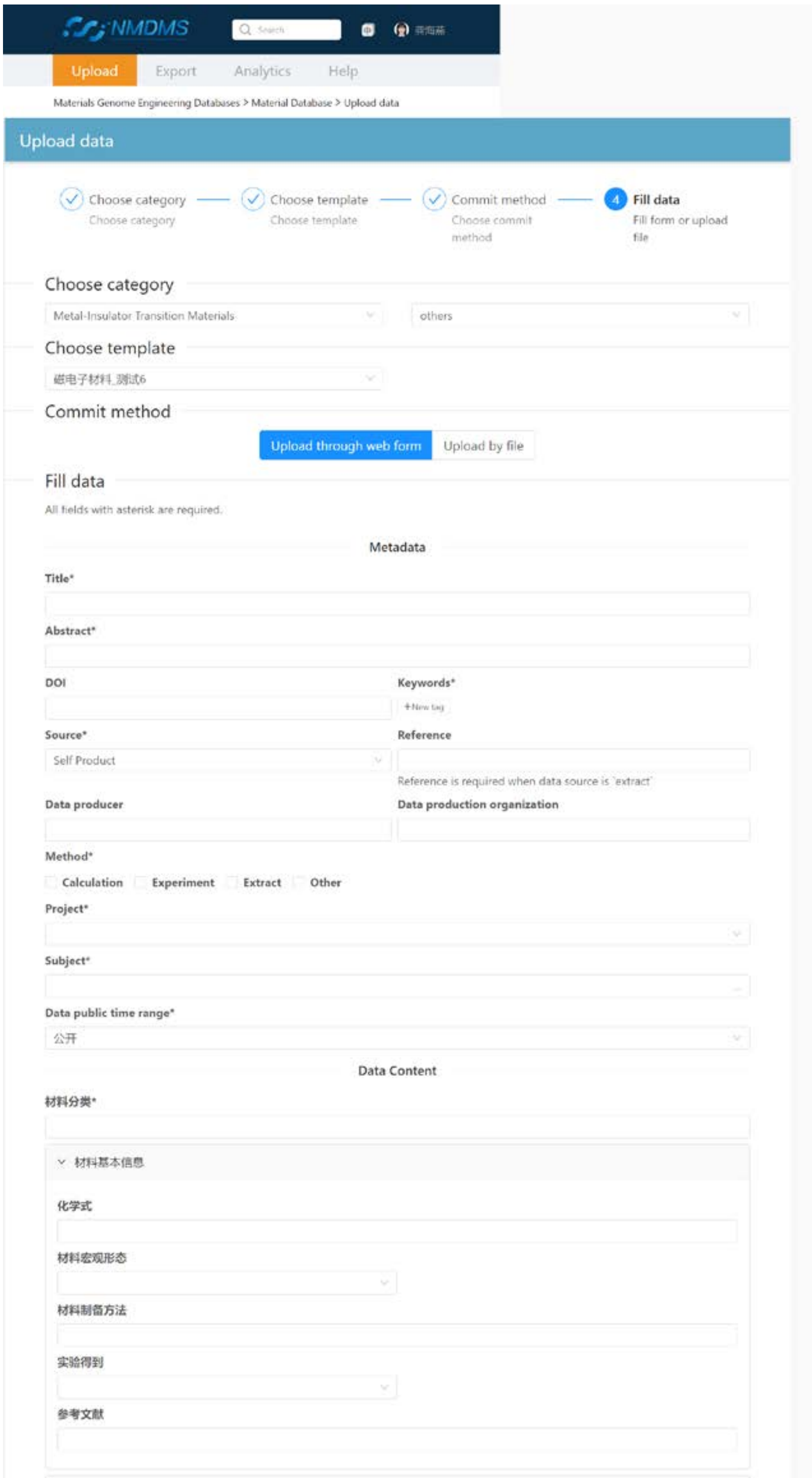

Data schema creation

If you have the permission of “模板上传（schema submission）”, you could create your own data schema. As shown below, choose the Template below “Upload” button, we can enter the page of creating data schema.

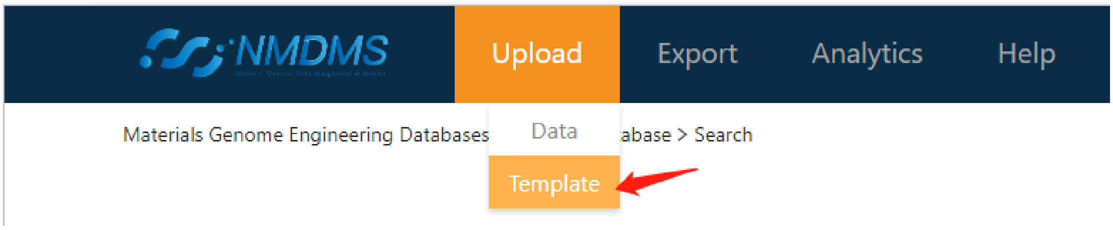

After entering the data schema creation module, you could choose to “upload template snippet” or “upload Data Template”. The template snippet (or data schema snippet) can be reused by creating a standardized data schema.

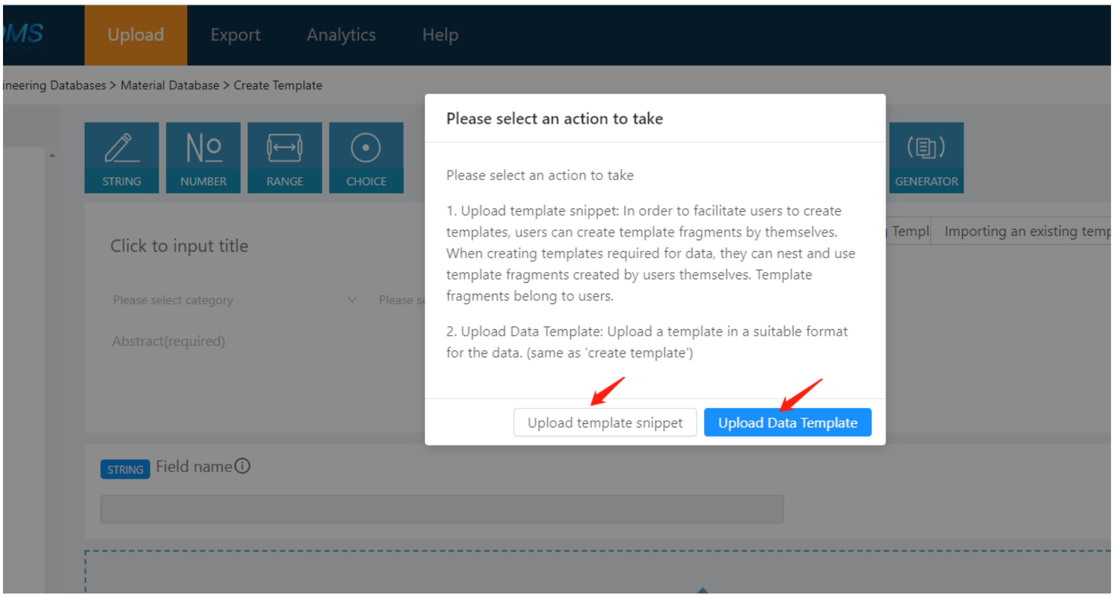

When create a data schema, we choose the “Importing an existing template” to import an existing data schema.

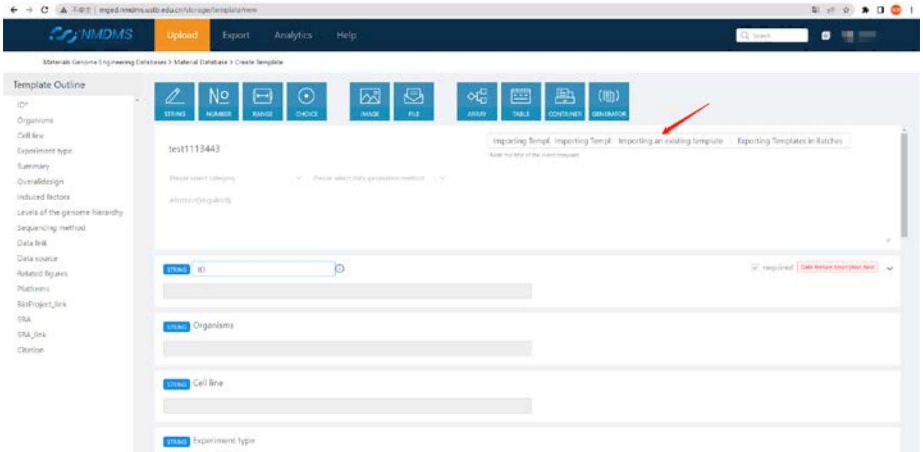

More information about creating a dynamic container schema can be seen on YouTube https://www.youtube.com/watch?v=G0_54ST97Ns.

Analytics

By clicking on the Analytics button, we can see the Analytics data according to materials categories or project in NMDMS platform.

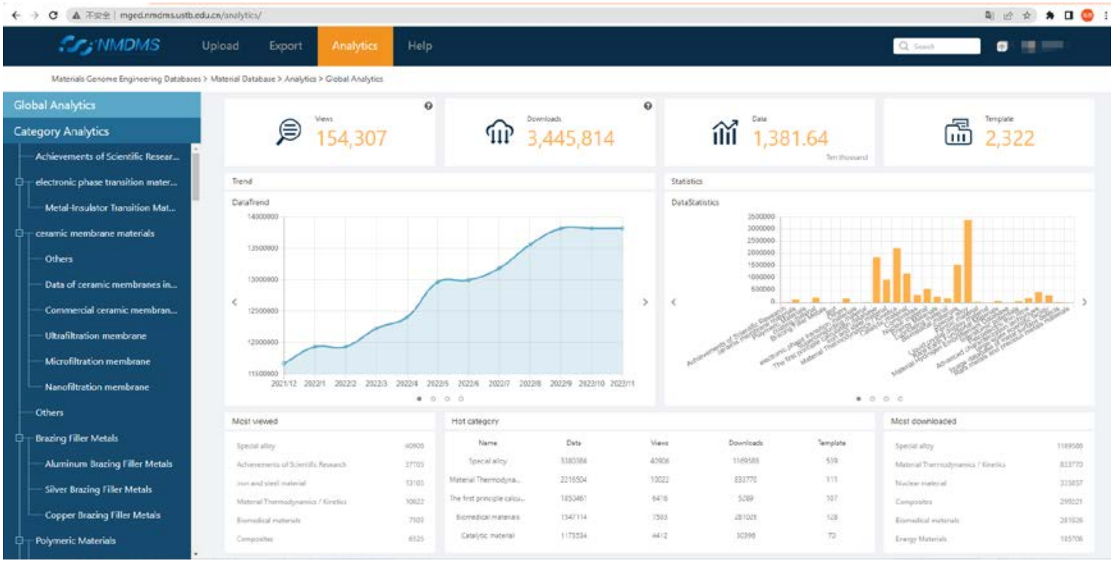

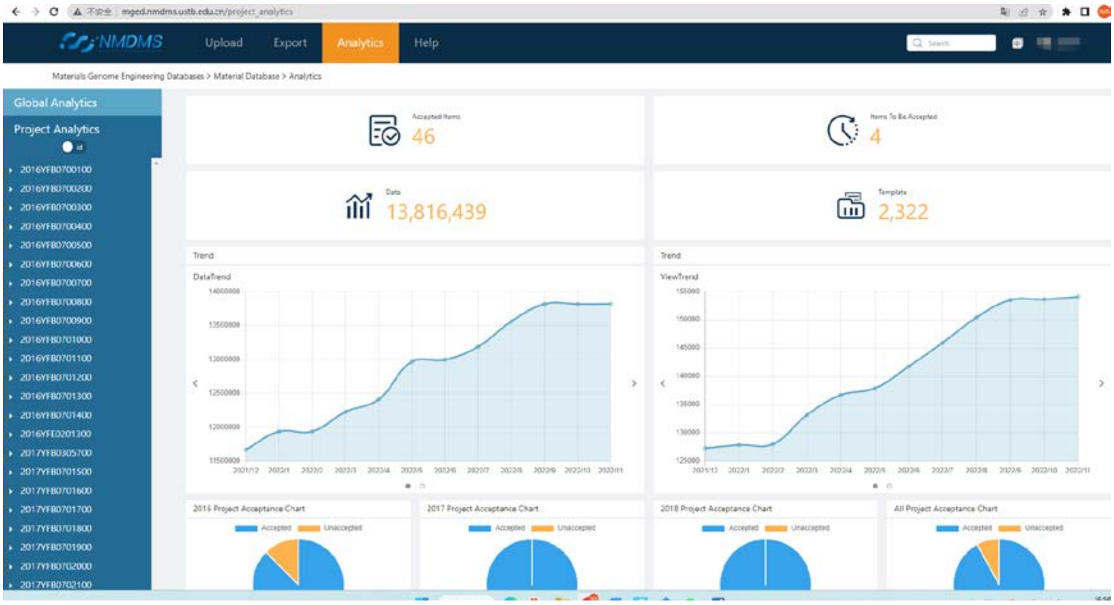

Data schema review

If you have the permission of “模板管理员（apply to be a reviewer for data schema）”,you can click on the button of “Review Templates” to review data schemas under some materials categories.

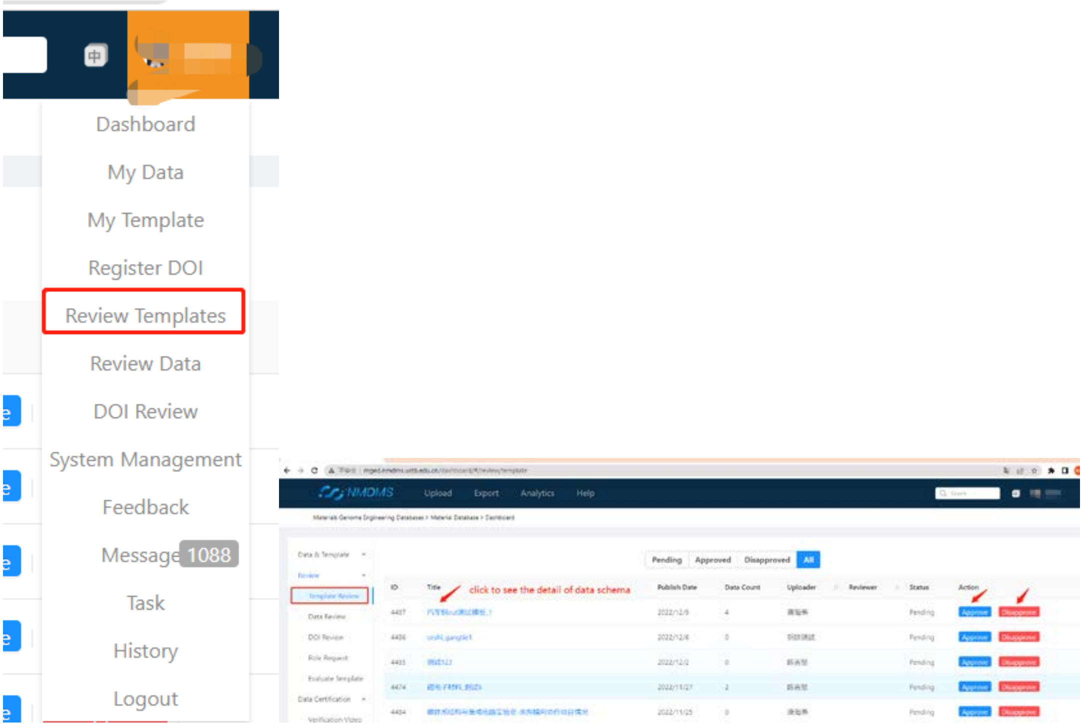

Data review

If you have the permission of “数据管理员（apply to be a reviewer for data）”, you can click on the button of “Review Data” to review data under some materials categories. You can choose approve or disapprove the data after viewing data.

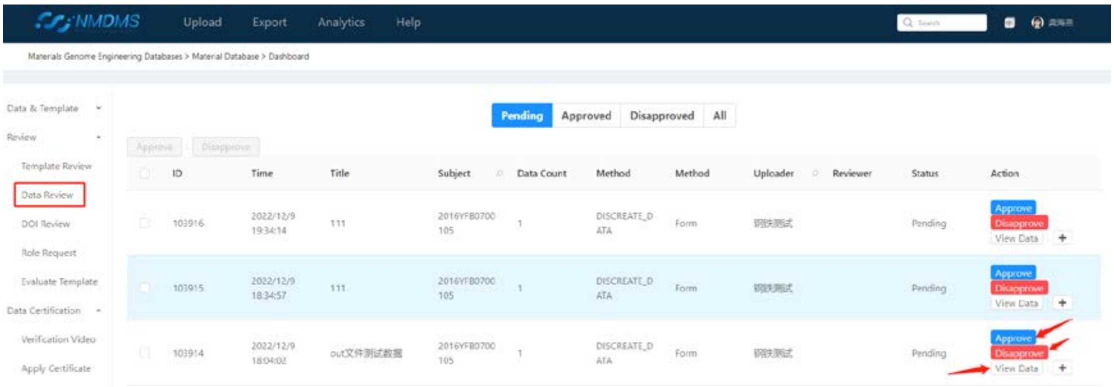

Data certification

Data certification includes “apply certificate”, “apply verification”, distribute verification”, “Evaluate verification” and “Certification data”.

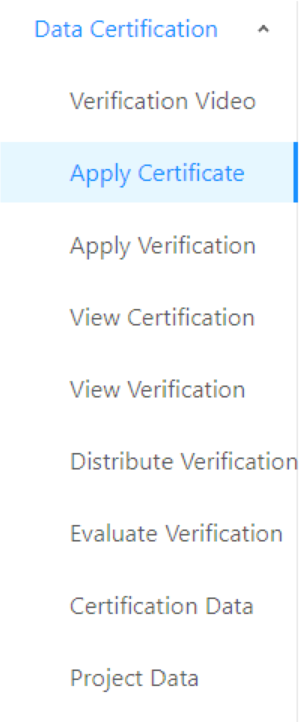

apply certificate:

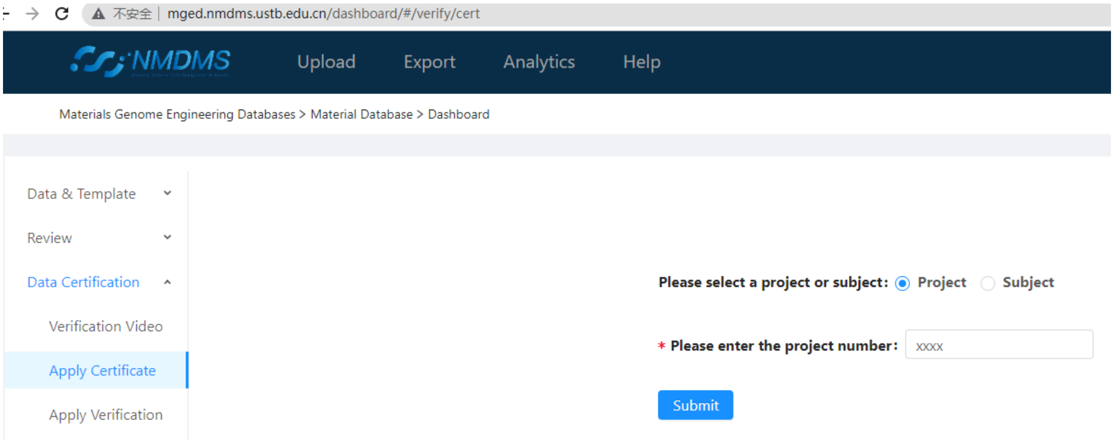

apply verification:

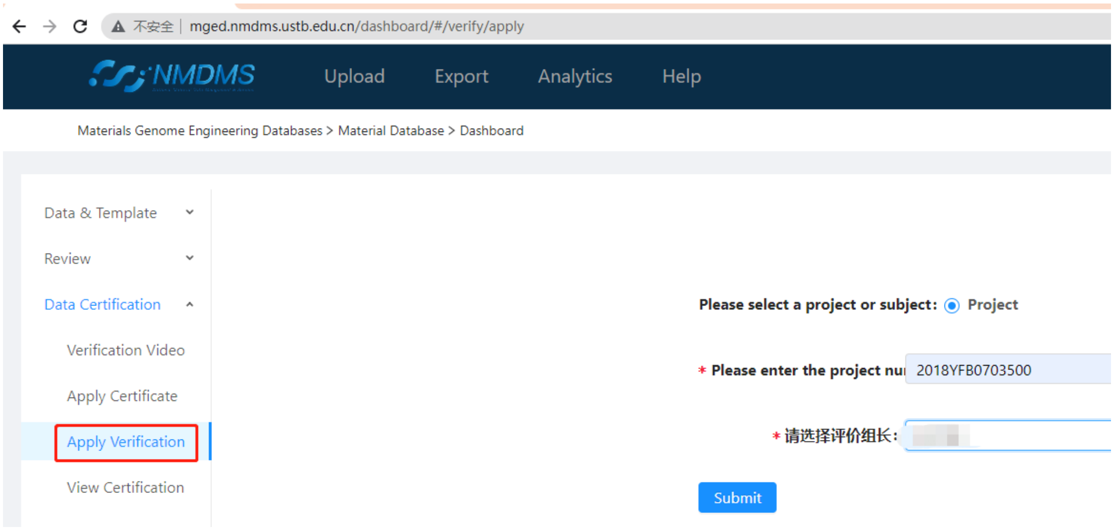

distribute verification:

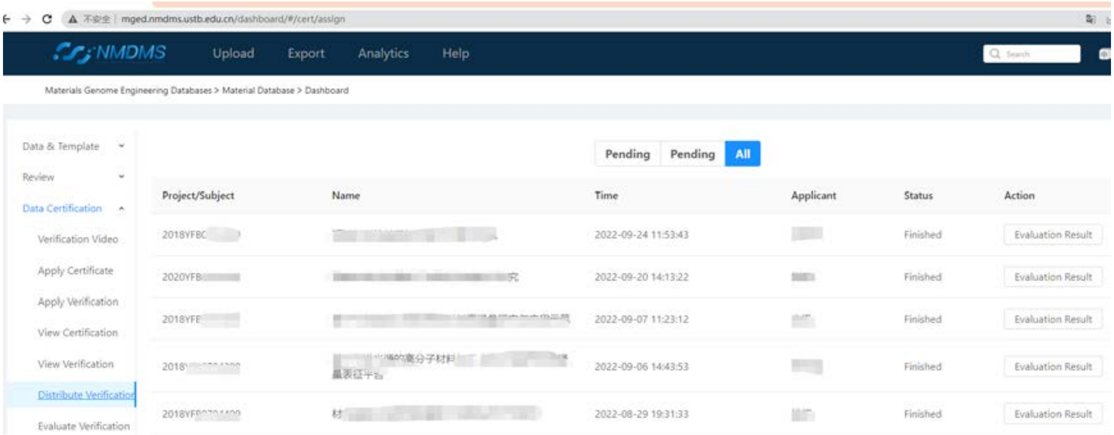

Evaluate verification:

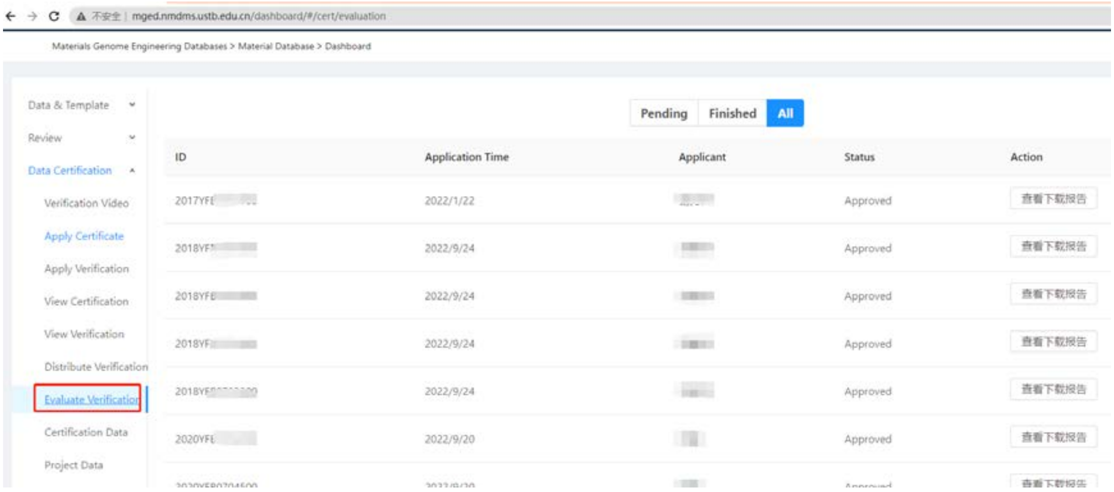

Certification data:

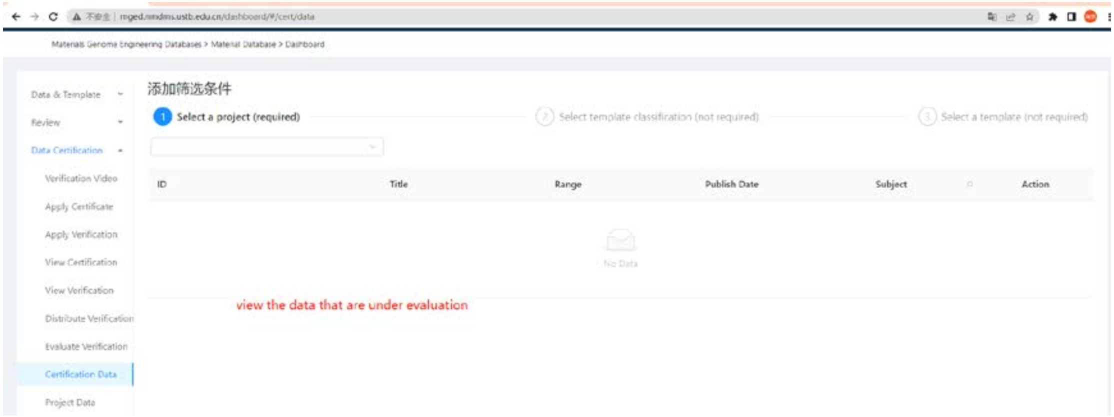

Register DOI

NMDMS also provides the registration of a digital object identifier (DOI) for dataset records. You can choose the button “Register DOI” to register a DOI for a dataset.

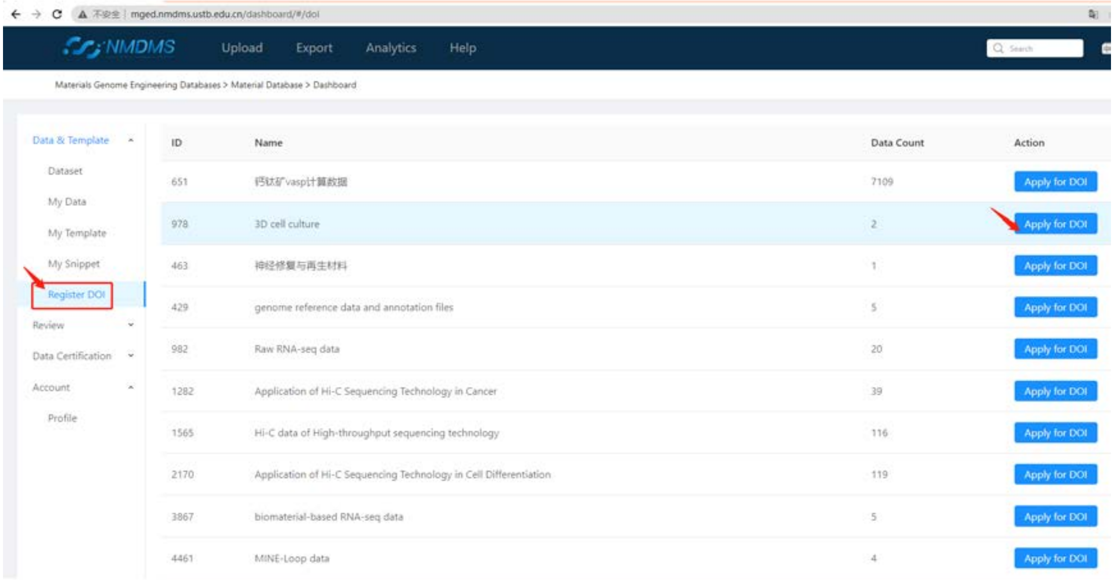

User manual for the relational database data submission module

By clicking on “关系型数据库数据汇交”, you can enter the page of “the relational database data submission module”.

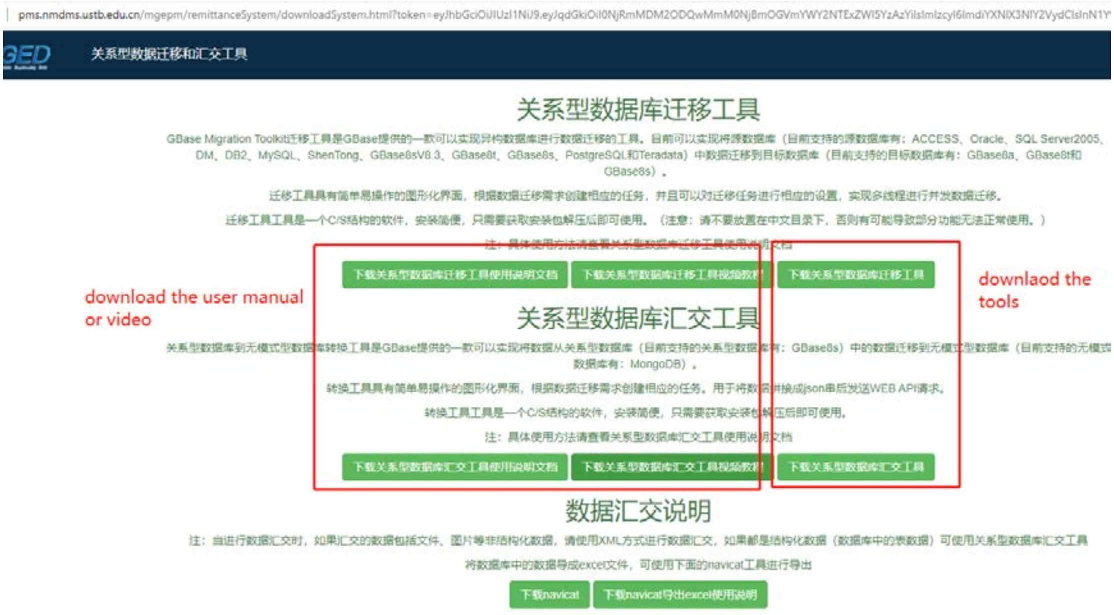

User manual for the high-throughput calculation data submission module

By clicking on “高通量计算数据汇交”, you can enter the page of “The High-throughput

calculation data submission module”. Choose the “算例管理（calculation data management）” , and choose “新增（add a new calculation data）”to add a new calculation data or “批量上传” to add multiple calculation dataset.

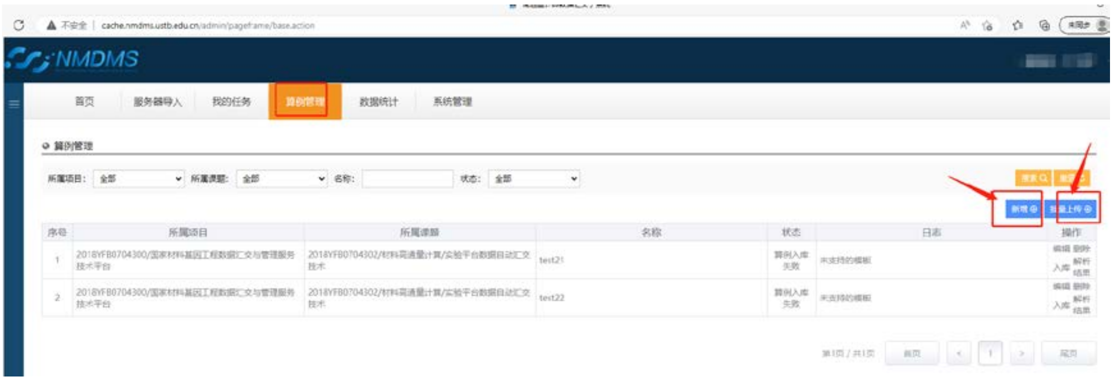

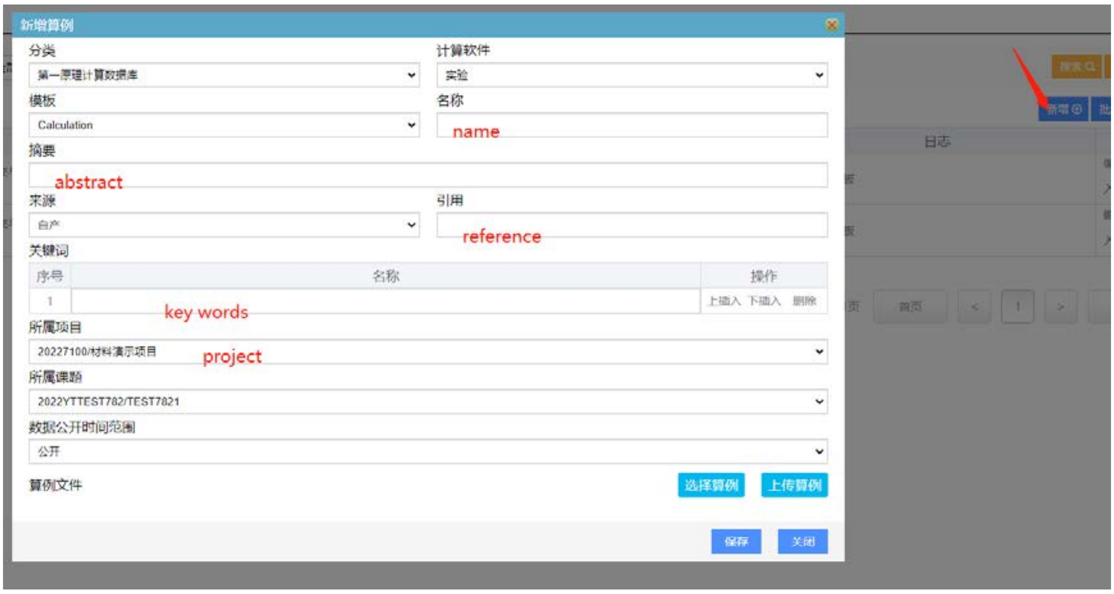

User manual for the experiment device data submission module

By clicking on “大科学装置实验数据汇交”, you can enter the page of “the experiment device data submission module”. Click on the “数据汇交”button to submit data. You can choose the button of “添加”to upload data on the webpage, or choose “批量上传” to upload data using a excel, where the excel template can be download by clicking on the button of “数据批量上传模板下载” .

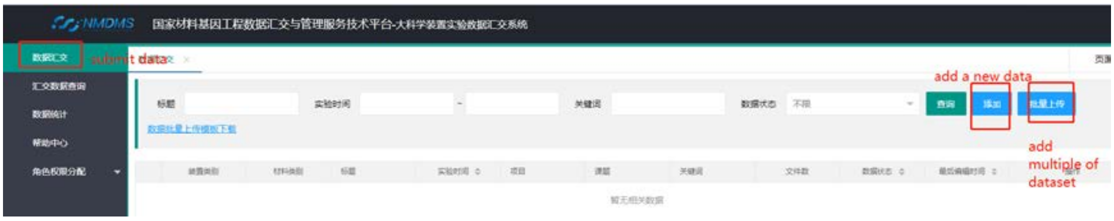

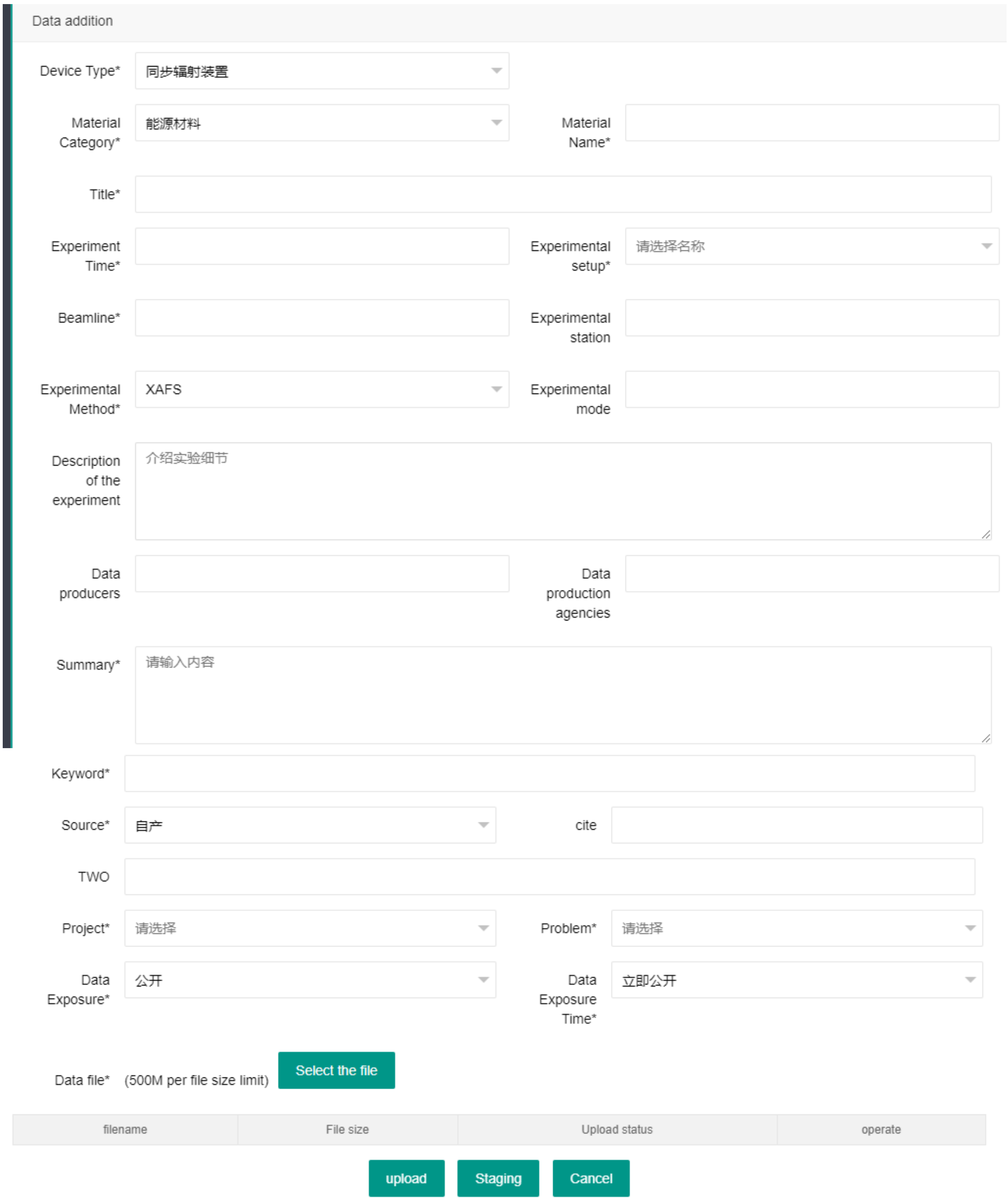
Data addition
Device Type*
同步辐射装置
Material Category*
能源材料
Material Name*
Title*
Experiment Time*
Experimental setup*
请选择名称
Beamline*
Experimental station
Experimental Method*
XAFS
Experimental mode
Description of the experiment
介绍实验细节
Data producers
Data production agencies
Summary*
请输入内容
Keyword*
Source*
自产
cite
TWO
Project*
请选择
Problem*
请选择
Data Exposure*
公开
Data Exposure Time*
立即公开
Data file*
(500M per file size limit)
Select the file
filename
File size
Upload status
operate
upload
Staging
Cancel
